# Preoperative *APOE* and Alzheimer’s disease polygenic risk profiling for perioperative neurocognitive disorders

**DOI:** 10.3389/fbinf.2026.1829278

**Published:** 2026-05-12

**Authors:** Mengquan Tan, Jiling Zeng, Huixian Zhou, Shilin Yang, Tong Wang, Meiling Zhong, Tongyu Wang, Zheng Liu, Yaling Dai, Siyuan Song

**Affiliations:** 1 Institute of Rehabilitation Industry, Fujian University of Traditional Chinese Medicine, Fuzhou, Fujian, China; 2 Department of Internal Medicine, Nazareth Hospital, Philadelphia, PA, United States; 3 Department of Internal Medicine, Montefiore Medical Center, Albert Einstein College of Medicine, New York, NY, United States; 4 Department of Neurology, Huashan Hospital, Fudan University, Shanghai, China; 5 Department of Biology, Duke University, Durham, NC, United States; 6 Department of General Practice, The Third People’s Hospital of Shenzhen, Shenzhen, Guangdong, China; 7 Shanghai Diabetes Institute, Shanghai Key Laboratory of Diabetes Mellitus, Shanghai Key Clinical Center for Metabolic Diseases, Shanghai Jiao Tong University Affiliated Sixth People’s Hospital, Shanghai, China; 8 Department of Pathology, The University of Texas MD Anderson Cancer Center, Houston, TX, United States; 9 Fujian Key Laboratory of Aptamers Technology, Fuzhou General Teaching Hospital (The 900th Hospital), Fujian University of Traditional Chinese Medicine, Fuzhou, Fujian, China; 10 Department of Anesthesiology, Montefiore Medical Center, Albert Einstein College of Medicine, Bronx, NY, United States; 11 Department of Neuroscience, Baylor College of Medicine, Houston, TX, United States

**Keywords:** Alzheimer’s disease polygenic risk score, APOE, blood-brain barrier, microglia, neuroinflammation, perioperative neurocognitive disorders, postoperative delirium, risk stratification

## Abstract

Perioperative neurocognitive disorders (PND) include postoperative delirium within 7 days after surgery, delayed neurocognitive recovery up to 30 days, and postoperative neurocognitive disorder up to 12 months. These outcomes are related, but they are not the same. They arise from the interaction of baseline brain vulnerability and perioperative stress, including inflammation, vascular instability, blood–brain barrier injury, metabolic strain, and reduced neural reserve. Preoperative genetic profiling is useful because it can estimate latent susceptibility before surgery. Among current signals, *APOE* is the strongest and most biologically relevant locus. At the same time, Alzheimer’s disease polygenic risk scores (AD-PRS) can capture non-*APOE* common-variant burden across lipid transport, endosomal trafficking, innate immune signaling, complement activity, microglial regulation, mitochondrial stress, and neurovascular integrity. Recent perioperative cohort studies have begun to test preoperative APOE-based and polygenic neurocognitive risk in surgical patients. Large delirium genetics studies also show a strong signal at the *APOE* locus and support overlap between delirium risk and Alzheimer’s disease-related common-variant architecture. These findings support an APOE-aware framework in which *APOE* genotype is modeled separately from non-*APOE* AD-PRS. In clinical use, this genomic layer should be combined with baseline cognition, frailty, vascular comorbidity, surgery-related risk, and circulating biomarkers such as neurofilament light chain. This review summarizes the loci, molecular pathways, and translational model designs that can move preoperative genomic profiling from association to perioperative risk stratification.

## 1 Introduction

Older adults now account for a large and growing share of the surgical population, and perioperative neurocognitive disorders (PND) have become an important clinical problem in this group (Evered et al., 2018a; Ren et al., 2025; Paternò et al., 2026). These disorders are associated with longer hospital stay, greater care burden, loss of independence, and worse longer-term cognitive outcomes (Evered et al., 2018a; Ren et al., 2025; Paternò et al., 2026). The problem is especially important in aging surgical populations because many patients already have reduced cognitive reserve, frailty, vascular comorbidity, sleep disturbance, or unrecognized neurodegenerative vulnerability before surgery (Evered et al., 2018a; Ren et al., 2025; Paternò et al., 2026). In this setting, surgery and anesthesia do not act on a uniformly healthy brain. They act on a brain with variable baseline resilience.

In this review, we use the 2018 perioperative nomenclature throughout. We use PND as the umbrella term, and we reserve postoperative delirium, delayed neurocognitive recovery, and postoperative neurocognitive disorder for their specific clinical contexts. We avoid the legacy term postoperative cognitive dysfunction except when referring to older literature. These phenotypes are related, but they are not identical (Evered et al., 2018a; Ren et al., 2025; Paternò et al., 2026; Leung et al., 2007; Vasunilashorn et al., 2015; Vasunilashorn et al., 2020).

Postoperative delirium is an acute syndrome marked by inattention, fluctuating awareness, and rapid changes in mental status. Delayed neurocognitive recovery and postoperative neurocognitive disorder are more often characterized by impairment in memory, executive function, attention, or overall cognitive performance (Leung et al., 2007; Vasunilashorn et al., 2015; Vasunilashorn et al., 2020). When available, this estimate may be further improved by considering baseline phenotype, early neuropsychiatric features, and biomarker evidence of active neurodegenerative stress.

Perioperative neurocognitive decline should also be viewed as a dynamic transition rather than a single static postoperative event. In older adults, baseline clinical phenotypes and early neuropsychiatric symptoms may reflect a brain that is already moving along a neurodegenerative trajectory before surgery. Recent cerebrospinal fluid proteomics studies in the mild cognitive impairment–Alzheimer’s disease continuum support this concept and suggest that genetic susceptibility is likely to interact with baseline phenotype and biomarker-defined biology, rather than act in isolation. This principle is relevant to perioperative screening, where surgical stress may unmask latent neurocognitive vulnerability (Han et al., 2026; Vromen et al., 2022).

In this setting, current perioperative risk assessment already identifies several important predictors, including age, frailty, baseline cognitive impairment, cerebrovascular disease, systemic inflammation, and the magnitude of surgical stress (Evered et al., 2018a; Ren et al., 2025; Paternò et al., 2026; Riaz et al., 2021; Kertai et al., 2021; Clark et al., 2022; Tang et al., 2024). However, these factors do not fully explain why patients with apparently similar clinical profiles can show very different postoperative neurocognitive outcomes. This gap is especially relevant in older adults, in whom subtle preclinical brain vulnerability may not be captured by routine perioperative assessment alone. In this setting, polygenic risk stratification is best understood as a way to estimate latent susceptibility before the perioperative insult occurs. It is not a stand-alone diagnostic test. Its value lies in its ability to add genomic information to established clinical variables and to refine preoperative estimates of neurocognitive vulnerability (Riaz et al., 2021; Kertai et al., 2021; Clark et al., 2022; Tang et al., 2024).

**FIGURE 1:**
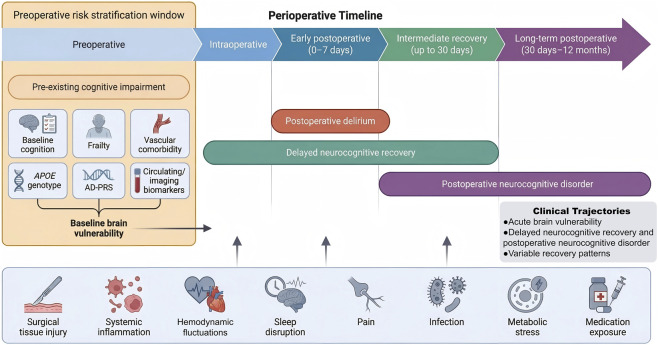
Clinical framework of perioperative neurocognitive disorders and the preoperative window for genomic risk stratification. This schematic shows the perioperative timeline across the preoperative, intraoperative, early postoperative (0–7 days), intermediate recovery (up to 30 days), and long-term postoperative (30 days-12 months) phases. It also places the main clinical phenotypes within perioperative neurocognitive disorders (PND) along this timeline. Pre-existing cognitive impairment is shown as an important baseline condition before surgery. Postoperative delirium, delayed neurocognitive recovery, and postoperative neurocognitive disorder are placed according to their usual time windows after surgery. Below the timeline, major perioperative stressors are shown, including surgical tissue injury, systemic inflammation, hemodynamic fluctuations, sleep disruption, pain, infection, metabolic stress, and medication exposure. These factors act together and increase brain vulnerability across the perioperative course. The highlighted preoperative risk stratification window shows the opportunity to combine baseline cognition, frailty, vascular comorbidity, *APOE* genotype, Alzheimer’s disease polygenic risk score (AD-PRS), and circulating or imaging biomarkers before surgery to identify patients at higher risk. These factors shape acute brain vulnerability, postoperative delirium, delayed neurocognitive recovery, postoperative neurocognitive disorder, and different postoperative recovery patterns. The figure provides the clinical basis for preoperative genomic risk assessment in patients at risk for PND.

Among current genetic signals, *APOE* remains the strongest and most interpretable locus linked to perioperative neurocognitive risk (Thedim et al., 2025; Raptis et al., 2026; Yang et al., 2025). Still, a single-locus model is unlikely to capture the full range of biologic susceptibility. A broader approach uses Alzheimer’s disease-informed polygenic risk scores together with dementia-related loci to estimate background neurodegenerative and neuroinflammatory liability. This approach is relevant because perioperative neurocognitive decline may reflect the unmasking of pre-existing vulnerability rather than a wholly new disease process (Paternò et al., 2026; Thedim et al., 2025; Thedim et al., 2026). Recent perioperative biobank studies and large-scale delirium genetics support the potential relevance of *APOE*-aware and Alzheimer’s disease-informed genomic profiling in perioperative neurocognitive risk, although the current evidence remains stronger for association than for direct improvement over clinical-only prediction models (Thedim et al., 2025; Raptis et al., 2026; Thedim et al., 2026) (Figure 1).

In this review, we examine how *APOE* and Alzheimer’s disease-related polygenic burden may inform perioperative neurocognitive risk, how these signals may interact with baseline phenotype and biomarker-defined vulnerability, how they map onto molecular pathways relevant to surgery and recovery, and how a clinically useful perioperative genomic framework might be designed.

## 2 *APOE* as a core locus in perioperative neurocognitive risk

*APOE* is located on chromosome 19 and encodes a 299-amino-acid apolipoprotein that is central to lipid and cholesterol transport in the brain (Husain et al., 2021; Budny et al., 2025; Al-Ghraiybah et al., 2026). The three main human isoforms, *APOE2*, *APOE3*, and *APOE4*, are defined by amino acid differences at residues 112 and 158. *APOE2* carries cysteine at both sites, *APOE3* carries cysteine at position 112 and arginine at position 158, and *APOE4* carries arginine at both sites. These small structural differences change the shape, stability, and lipid-binding behavior of the protein. They also change how *APOE* interacts with cell-surface receptors and lipoprotein particles (Husain et al., 2021; Troutwine et al., 2022; Islam et al., 2025). In practical terms, the isoforms do not function in the same way in neural tissue, and *APOE4* is the form most strongly linked to late-onset alzheimer’s disease risk (Budny et al., 2025; Al-Ghraiybah et al., 2026).

In the central nervous system, astrocytes are the main source of *APOE* under normal conditions. Microglia also produce *APOE*, and this expression increases in disease-related and activated states (Al-Ghraiybah et al., 2026; Lin and Holtzman, 2024; Strickland et al., 2026). After synthesis, *APOE* is secreted as part of lipoprotein particles and binds members of the *LDL* receptor family, including *LDLR* and *LRP1*. Through these receptor systems, *APOE* helps control lipid redistribution between glial cells and neurons, membrane turnover, synaptic maintenance, and the clearance of cellular debris (Troutwine et al., 2022; Liu et al., 2025). This places *APOE* in a central position in brain homeostasis. It is not only a marker of neurodegenerative risk. It is part of the machinery that supports membrane repair and metabolic balance in aging neural tissue.

The relevance of *APOE* to perioperative neurocognitive disorders comes from the fact that the same biologic systems regulated by *APOE* are stressed during surgery and in the postoperative period. One major mechanism is amyloid-β handling. *APOE* binds amyloid-β and influences its aggregation, deposition, and clearance (Al-Ghraiybah et al., 2026; Xia et al., 2024). Another major mechanism is immune regulation. *APOE* shapes microglial activation and inflammatory signaling, in part through receptor-linked pathways that affect downstream kinase activity and cytokine response (Lin and Holtzman, 2024; Strickland et al., 2026). *APOE* also affects synaptic lipid supply and membrane composition, which can alter how well synapses maintain transmission during acute systemic stress (Islam et al., 2025; Liu et al., 2025). In older adults, these effects can be especially important because baseline synaptic reserve is already reduced, and the brain may be less able to compensate for inflammation, sleep disruption, metabolic instability, and medication exposure after surgery (Thedim et al., 2026).

A second major mechanism is blood–brain barrier integrity. Experimental studies show that *APOE4* is linked to a more permeable barrier phenotype than *APOE3* (Kirchner et al., 2023). In *APOE4* models, blood–brain barrier dysfunction has been associated with increased *MMP9*, degradation of tight junction proteins such as ZO-1, occludin, and claudin-5, and reduced astrocytic end-foot coverage of cerebral vessels (Kirchner et al., 2023; Chen et al., 2024). *APOE4* is also linked to weaker control of the pericyte *CYPA*-*MMP9* pathway, which promotes vascular injury over time (Kirchner et al., 2023). These vascular effects are directly relevant to perioperative cognition because surgery can increase systemic inflammation, endothelial stress, and hemodynamic instability (Thedim et al., 2026). In a brain with reduced vascular resilience, these perioperative insults may more easily lead to acute attention deficits, fluctuating cognition, and slower cognitive recovery.

## 3 Current perioperative genetic evidence

The strongest current common-variant signal linked to delirium comes from a large multi-ancestry genome-wide association study and meta-analysis (Raptis et al., 2026). That study included 1,059,130 individuals, including 11,931 delirium cases, from the United Kingdom Biobank, FinnGen, All of Us Research Program, and Michigan Genomics Initiative cohorts. The lead signal was rs429358 in *APOE*, with an odds ratio of 1.60 (95% CI 1.55–1.65; P = 9.7 × 10^−177^). The linked rs7412 signal was also significant (OR 0.84, 95% CI 0.79–0.88; P = 1.8 × 10^−11^). These two coding variants are especially important because they define the classic *APOE* ε2, ε3, and ε4 haplotypes (Raptis et al., 2026; Belloy et al., 2019). This means that the delirium association maps directly to the core *APOE* coding structure rather than to a nearby noncoding marker that only tracks with the locus through linkage disequilibrium (Raptis et al., 2026). In practical terms, this provides more direct genetic evidence for *APOE* involvement in delirium (Sepulveda et al., 2021). It also places the main delirium signal within the same *APOE* framework that is already central to late-life neurodegenerative risk (Raptis et al., 2026; Lake et al., 2023; Corder et al., 1993; Kunkle et al., 2019; Jansen et al., 2019).

The same study also showed that the *APOE* signal was not explained only by pre-existing dementia. After adjustment for dementia status, rs429358 remained significantly associated with delirium (OR 1.20, 95% CI 1.12–1.28; P = 3.7 × 10^−15^). The signal also remained significant in dementia-free cohorts (OR 1.27, 95% CI 1.20–1.34; P = 4.7 × 10^−18^) and in Alzheimer’s disease-free cohorts (OR 1.45, 95% CI 1.38–1.52; P = 3.9 × 10^−56^) (Raptis et al., 2026). These findings suggest that *APOE* may reflect a broader form of acute brain vulnerability under stress, and not only background dementia liability. In perioperative terms, the *APOE* signal in delirium may capture reduced brain resilience under stress rather than only background dementia risk (Thedim et al., 2025; Raptis et al., 2026; Corder et al., 1993).

The same analysis also helped separate the main effect within the chromosome 19 region. When the model was conditioned on *APOE* ε4 haplotype count, the strong *APOE*-region association largely disappeared. This supports the view that *APOE* ε4 is the main independent driver of the signal in that region. After this conditioning step, additional loci became easier to detect. These included an intronic variant in *ADAM32* (rs531178459) and, in sensitivity analyses, signals near *SEC14L1* (Raptis et al., 2026). The investigators also used knowledge-based prioritization methods and highlighted several genes within the broader chromosome 19 interval, including *APOE*, *TOMM40*, *PVRL2*, and *BCAM* (Raptis et al., 2026). This is useful because it shows that the local signal is not biologically simple. Even though *APOE* is the dominant association, it sits in a dense genomic neighborhood that includes genes linked to mitochondrial function, membrane biology, vascular interactions, and cellular transport (Belloy et al., 2019; Roses et al., 2010). This local structure matters when the region is interpreted in translational models, since some nearby genes may influence perioperative brain stress responses even if they do not carry the strongest independent association.

The same delirium genetics work also supported a shared genetic background between delirium and Alzheimer’s disease (Raptis et al., 2026; Deiner and Silverstein, 2009). The investigators used summary statistics from large Alzheimer’s disease genome-wide studies and found that AD-linked information could improve discovery in delirium analyses (Kunkle et al., 2019; Jansen et al., 2019). This supports the idea that part of delirium risk is connected to the same common-variant architecture that shapes late-life neurodegenerative vulnerability (Deiner and Silverstein, 2009; Davis et al., 2012). Within this shared framework, several non-*APOE* genes were highlighted as important targets for future work, including *MS4A4A*, *CR1*, and *TOMM40* (Roses et al., 2010; Lambert et al., 2013). These genes are notable because they are linked to biologic systems that are highly relevant to perioperative neurocognitive injury, such as microglial signaling, complement-related immune activity, and mitochondrial stress (Paternò et al., 2026; Yang et al., 2025; Hollingworth et al., 2011; Crehan et al., 2012; Shi et al., 2017). This makes them useful candidates for pathway-based models, especially if future studies move from single-locus association toward Alzheimer’s disease-informed polygenic risk scores or pathway-restricted scores built around immune, vascular, and membrane-trafficking networks (Thedim et al., 2025; Kunkle et al., 2019; Jansen et al., 2019; Deiner and Silverstein, 2009).

The perioperative literature is smaller and less consistent. In an early nested cohort study of 190 patients aged 65 years or older undergoing major noncardiac surgery, 15.3% developed delirium on postoperative days 1 and 2, and one *APOE*-ε4 allele was associated with a higher adjusted risk of early postoperative delirium (OR 3.64, 95% CI 1.51–8.77) (Leung et al., 2007). In contrast, a later study of 557 nondemented patients aged 70 years or older undergoing major noncardiac surgery found no association between *APOE*-ε4 carrier status and postoperative delirium incidence (RR 1.0, 95% CI 0.7–1.5), severity, or duration (Vasunilashorn et al., 2015). A further analysis in 553 noncardiac surgical patients showed that *APOE*-ε4 did not act as a clear main-effect predictor, but it modified the association between postoperative day 2 CRP and delirium. Among *APOE*-ε4 carriers, high CRP was associated with increased delirium incidence (RR 3.0, 95% CI 1.4–6.7), whereas no significant association was observed in non-carriers (Vasunilashorn et al., 2020). Taken together, these perioperative cohort studies suggest that *APOE* is biologically relevant, but the observed effect depends on the cohort, endpoint, and analytic framework used.

More recent perioperative biobank work has moved the field closer to direct preoperative genetic risk profiling in surgical populations (Clark et al., 2022). In the Mass General Brigham Biobank study of 33,526 surgical patients aged 40–89 years without previous Alzheimer’s disease, 86% of participants were of European ancestry. Among patients of European ancestry, *APOE*-ε4 carriage was associated with higher odds of delirium (OR 1.32, 95% CI 1.19–1.47), mild cognitive impairment (OR 1.70, 95% CI 1.49–1.94), and Alzheimer’s disease (OR 3.42, 95% CI 2.72–4.29), while the Alzheimer’s disease polygenic risk score was associated with higher odds of Alzheimer’s disease (OR 2.25, 95% CI 1.64–3.09) (Clark et al., 2022). These findings support the feasibility of perioperative genomic profiling in real-world surgical cohorts, but they mainly support association rather than incremental prediction beyond clinical variables.

Evidence for prediction gain remains limited. In the delirium genetic and proteomic study by Raptis et al., the proteomic analysis included 32,652 United Kingdom Biobank participants, including 541 incident delirium cases. Adding 18 selected proteins to a basic model that included age, sex, and BMI increased the AUC from 0.764 to 0.791, but this increase was not significant (P = 0.09). When *APOE*-ε4 status was added together with the proteins, the AUC increased from 0.764 to 0.794, and this improvement was significant (P = 0.049) (Raptis et al., 2026). This result suggests modest but measurable improvement beyond a simple non-genetic model. However, comparable model-comparison analyses remain limited in perioperative cohorts with clearly defined postoperative endpoints (Table 1).

**TABLE 1:** Key studies on APOE and polygenic risk in perioperative neurocognitive disorders.

Reference	Study design/ Population	Sample size	Endpoint(s)	Main genetic variable	Main quantitative finding	Increment beyond clinical-only model
Leung et al. (2007)	Nested cohort; patients ≥65 years undergoing major noncardiac surgery	190	Early postoperative delirium on postoperative days 1–2	*APOE*-ε4 carrier status	Adjusted OR 3.64, 95% CI 1.51–8.77	Not reported
Vasunilashorn et al. (2015)	Prospective cohort; nondemented patients ≥70 years undergoing major noncardiac surgery	557	Delirium incidence, severity, duration	*APOE*-ε2/ε4 carrier status	ε4 not associated with delirium incidence; RR 1.0, 95% CI 0.7–1.5	Not reported
Vasunilashorn et al. (2020)	Prospective cohort; noncardiac surgical patients ≥70 years	553	Delirium incidence, severity, duration	*APOE*-ε4 × postoperative CRP	In ε4 carriers, high CRP associated with delirium incidence; RR 3.0, 95% CI 1.4–6.7	Not reported
Thedim et al. (2025)	Surgical biobank study; patients aged 40–89 years without prior AD	33,526	Delirium, MCI, AD	*APOE*-ε4; AD-PRS	Delirium OR 1.32, 95% CI 1.19–1.47; MCI OR 1.70, 95% CI 1.49–1.94; AD OR 3.42, 95% CI 2.72–4.29; AD-PRS and AD OR 2.25, 95% CI 1.64–3.09	Not clearly established in perioperative model comparison
Raptis et al. (2026)	Multi-ancestry GWAS/meta-analysis using United Kingdom biobank, FinnGen, all of us, and Michigan genomics Initiative	1,059,130 total participants; 11,931 delirium cases	Delirium genetic association	*APOE* rs429358 (lead variant)	rs429358 was the lead delirium signal: OR 1.60, 95% CI 1.55–1.65; after dementia adjustment, the association remained significant: OR 1.20, 95% CI 1.12–1.28	Not applicable/not reported as a clinical prediction model comparison
Raptis et al. (2026)	United Kingdom biobank European-ancestry proteomic cohort; PWAS plus prediction modeling for incident delirium	32,652 participants; 541 incident delirium cases	Incident delirium prediction (up to 16 years of follow-up)	*APOE*-ε4 status+18 stability-selected plasma proteins	109 of 2,919 plasma proteins were associated with incident delirium; prediction models were built using age, sex, BMI, 18 stability-selected proteins, and *APOE*-ε4	Adding proteins + *APOE*-ε4 to the basic model (age, sex, BMI) improved AUC from 0.764 to 0.794 (P = 0.049)

## 4 Alzheimer’s disease polygenic risk beyond *APOE*

A perioperative neurocognitive model that includes only *APOE* can capture an important part of baseline genetic vulnerability, but it does not represent the full common-variant structure linked to late-life brain susceptibility (Thedim et al., 2025; Skoog et al., 2021). Alzheimer’s disease genetics now includes many validated common-risk loci outside the *APOE* region (Andrews et al., 2023; Belloy et al., 2023). These loci do not act through a single pathway. They cluster across several biologic systems that are also relevant to perioperative brain stress. These systems include lipid transport, innate immune signaling, complement activity, endosomal and lysosomal trafficking, membrane protein recycling, amyloid precursor protein handling, cytoskeletal stability, synaptic maintenance, and mitochondrial stress responses (Andrews et al., 2023; Chandler et al., 2025; Xu et al., 2024). Because of this, an Alzheimer’s disease polygenic risk score, or AD-PRS, is useful in perioperative research as a way to capture distributed baseline neurobiological risk that cannot be explained by *APOE* alone (Najar et al., 2023; Hou et al., 2024).

From a computational perspective, AD-PRS construction involves several steps that can materially affect score performance. Investigators must first select the discovery GWAS, because the size of the training dataset, case definition, ancestry composition, and variant-level quality control of the source study all influence the effect estimates used for score construction (Clark et al., 2022; Jiang et al., 2024; Privé et al., 2021; Ge et al., 2019). They must then harmonize summary statistics with the target dataset, including genome build, SNP identifiers, effect alleles, and strand orientation, and remove variants with poor imputation quality, very low frequency, duplication, or ambiguous alignment when needed (Clark et al., 2022; Ge et al., 2019). After this preprocessing step, SNP weights are assigned by the PRS algorithm. Early Alzheimer’s disease studies often used clumping-and-thresholding approaches, which prune correlated variants and retain SNPs according to predefined association P-value thresholds (Clark et al., 2022). This method is simple and transparent, but it may discard part of the polygenic signal. By contrast, newer linkage disequilibrium-aware methods, such as LDpred2 and PRS-CS, model correlation between nearby variants more directly and apply shrinkage to effect sizes across the genome (Jiang et al., 2024; Privé et al., 2021). These methods often yield more stable scores and can improve predictive performance, although their results still depend on the quality of the input GWAS and the analytic setting used (Jiang et al., 2024; Privé et al., 2021; Ge et al., 2019).

Several additional design choices are also important. Score performance depends on the linkage disequilibrium reference panel, the parameter-tuning strategy, and the ancestry match between the discovery dataset, the LD reference dataset, and the target cohort (Clark et al., 2022; Privé et al., 2021; Ge et al., 2019). In Alzheimer’s disease, handling of the APOE region is especially important because this locus has a very large effect and can dominate the overall score if it is left inside a genome-wide PRS without special treatment (Clark et al., 2022). For this reason, many studies separate *APOE* from the broader polygenic component and calculate a non-*APOE* AD-PRS after removing the extended APOE region (Clark et al., 2022; Xu et al., 2024; Sofer et al., 2023). In practical terms, perioperative studies should report the discovery GWAS, variant filtering and harmonization procedures, PRS algorithm, LD reference panel, tuning method, *APOE* handling strategy, and validation framework. They should also report whether the score improves discrimination, calibration, and risk stratification beyond clinical-only models (Clark et al., 2022; Ge et al., 2019). These details are important because two AD-PRS built from the same Alzheimer’s disease GWAS can perform differently if the computational pipeline differs.

In practical terms, *APOE* should still be modeled separately from the broader polygenic background. The *APOE* signal is stronger than most other common variants in Alzheimer’s disease genetics, and it can dominate a global score if it is not handled carefully (Belloy et al., 2023; Sofer et al., 2023; Fan et al., 2020). A perioperative model is therefore easier to interpret when it is built in two layers. The first layer is an *APOE*-aware component, which treats *APOE* genotype, especially ε4 carriage, as a distinct variable (Leung et al., 2007; Thedim et al., 2025). The second layer is a non-*APOE* AD-PRS, which is constructed from many common variants across validated Alzheimer’s disease loci outside the *APOE* region (Skoog et al., 2021; Xu et al., 2024; Najar et al., 2023; Sofer et al., 2023; Sampatakakis et al., 2024). This structure makes the model clearer in both biologic and statistical terms. It allows the clinician or researcher to distinguish between a patient whose risk is mainly driven by *APOE* and a patient whose risk is shaped by broader common-variant burden across multiple pathways. It also reduces the chance that the very large *APOE* effect will mask weaker but still meaningful signals from other loci (Xu et al., 2024; Sofer et al., 2023).

This non-*APOE* burden is biologically relevant because the additional loci point to mechanisms that may influence how the brain responds to surgery and anesthesia. For example, variants in *BIN1*, *PICALM*, *CD2AP*, and *SORL1* are linked to endocytosis, vesicle trafficking, and membrane recycling. These processes are important for synaptic function and receptor turnover (Andrews et al., 2023). Variants in *TREM2*, *CD33*, *INPP5D*, *PLCG2*, and *MS4A*-family genes are linked to microglial activation, phagocytic behavior, and inflammatory signaling (Andrews et al., 2023). These pathways are highly relevant to postoperative delirium, since delirium often develops in the setting of acute systemic inflammation and altered neuroimmune signaling (Leung et al., 2007; Li et al., 2025; Fournier et al., 2015). Variants in *CR1* point to complement-related immune activity, and variants near *TOMM40* point to mitochondrial transport and oxidative stress handling (Andrews et al., 2023; Chandler et al., 2025). Together, these loci support the view that non-*APOE* polygenic burden may reflect a broad state of reduced brain resilience, especially in older adults exposed to inflammatory, vascular, and metabolic stress during the perioperative period (Thedim et al., 2025; Supiyev et al., 2023; Thedim and Vacas, 2024).

**FIGURE 2:**
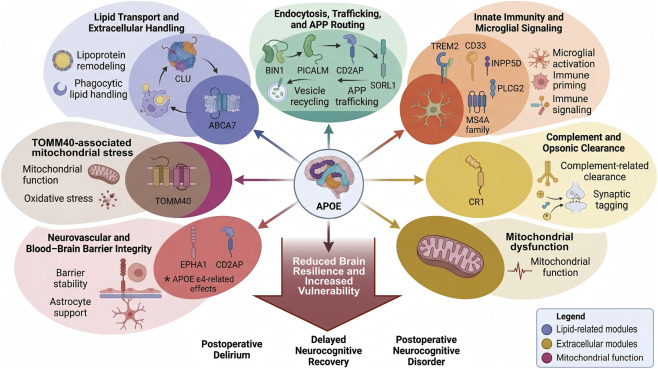
*APOE*-centered and non-*APOE* molecular networks relevant to perioperative neurocognitive vulnerability. This schematic shows an *APOE*-centered molecular framework that integrates major Alzheimer’s disease-related loci linked to perioperative neurocognitive vulnerability. *APOE* is placed at the center because it is involved in lipid transport, amyloid-β handling, microglial signaling, and blood–brain barrier integrity. Surrounding non-*APOE* risk genes are organized into major functional modules. The lipid transport and extracellular handling module includes *CLU* and *ABCA7*, which are linked to lipoprotein remodeling, extracellular chaperone activity, phagocytic lipid handling, and membrane repair support. The endocytosis, trafficking, and APP routing module includes *BIN1*, *PICALM*, *CD2AP*, and *SORL1*, which regulate endocytosis, vesicle recycling, endosomal sorting, amyloid precursor protein trafficking, and synaptic receptor turnover. The innate immunity and microglial signaling module includes *TREM2*, *CD33*, *INPP5D*, *PLCG2*, and the *MS4A* family, which influence microglial activation, immune signaling, and inflammatory state control. The complement and opsonic clearance module highlights *CR1*, which is linked to complement-related clearance, synaptic tagging, microglial recognition, and immune amplification. Mitochondrial stress and mitochondrial function are represented through *TOMM40*-associated and downstream bioenergetic pathways, with emphasis on mitochondrial protein import, oxidative stress, and cellular resilience. The neurovascular and blood–brain barrier integrity module includes *EPHA1*, *CD2AP*, and *APOE* ε4-related vascular effects, with emphasis on endothelial responses, barrier stability, astrocyte–vascular support, and permeability control. These pathways converge on reduced perioperative brain resilience and greater vulnerability to postoperative delirium, delayed neurocognitive recovery, and postoperative neurocognitive disorder. The figure provides an integrated molecular map linking *APOE* and non-*APOE* Alzheimer’s disease risk architecture to perioperative cognitive vulnerability.

There is already precedent for separating *APOE* from non-*APOE* polygenic risk in cognitive aging research. In older adults without baseline dementia, studies using small non-*APOE* polygenic panels have shown that higher non-*APOE* PRS is associated with a modest increase in dementia risk, while *APOE* ε4 produces a stronger and earlier effect (Skoog et al., 2021; Kauppi et al., 2020; Tomassen et al., 2022; Vasiljevic et al., 2023). This pattern is highly relevant to perioperative neurocognitive risk (Thedim et al., 2025; Najar et al., 2023). It suggests that *APOE* is likely to provide the clearest immediate signal of vulnerability, while the broader non-*APOE* score captures smaller, distributed effects that may become useful when combined with clinical variables such as age, frailty, baseline cognition, vascular burden, and surgery-related stress (Thedim et al., 2025; Hou et al., 2024; Supiyev et al., 2023; Loomis et al., 2024). In this setting, the value of AD-PRS is not that it replaces *APOE*. Its value is that it adds another layer of biologic information, especially when the goal is to estimate global neural vulnerability rather than to rely on one major locus alone (Thedim et al., 2025; Xu et al., 2024; Najar et al., 2023; Sampatakakis et al., 2024) (Figure 2).

## 5 Non-*APOE* loci and their functional molecular networks

### 5.1 Lipid transport and lipoprotein remodeling

Among non-*APOE* Alzheimer’s disease risk loci, *CLU* and *ABCA7* are closely linked to extracellular lipid handling, membrane maintenance, and cellular clearance (Duchateau et al., 2024; Zhao et al., 2025; Sprenger et al., 2025; Laslo et al., 2024). *CLU*, which encodes clusterin, is a secreted glycoprotein with chaperone-like properties (Laslo et al., 2024; Yuste-Checa et al., 2025). It binds lipids and misfolded proteins in the extracellular space, interacts with lipoprotein particles, and helps regulate protein solubility under stress conditions (Zhao et al., 2025). In neural tissue, clusterin is produced mainly by glial cells and participates in lipid redistribution, apoptosis-related signaling, and immune response pathways (Zhao et al., 2025; Milinkeviciute and Green, 2023). It also affects amyloid-β assembly and solubility, which links it to extracellular protein homeostasis around synapses and vessel walls (Laslo et al., 2024; Yuste-Checa et al., 2025). When *CLU*-linked function is weaker, damaged proteins and lipid-rich debris may persist longer in the extracellular environment, which can impair synaptic stability and increase local stress signaling (Yuste-Checa et al., 2025; Milinkeviciute and Green, 2023).

*ABCA7* encodes an ATP-binding cassette transporter that regulates phospholipid and cholesterol movement across cellular membranes (Duchateau et al., 2024; Sprenger et al., 2025; Santos-García et al., 2025). In the brain, *ABCA7* is strongly linked to membrane lipid balance and to phagocytic activity, especially in microglia (Duchateau et al., 2024; Sprenger et al., 2025; Santos-García et al., 2025; Aikawa et al., 2021). It contributes to the engulfment and clearance of cellular debris and has been associated with amyloid-β uptake and removal (Santos-García et al., 2025). Reduced *ABCA7* activity may weaken phagocytic clearance and disturb membrane composition, which in turn can affect receptor localization, vesicle fusion, and membrane repair (Duchateau et al., 2024; Sprenger et al., 2025). These effects are relevant in older adults because aging neurons and glial cells depend more heavily on efficient lipid turnover to maintain function under stress (Sprenger et al., 2025; He et al., 2025).

During the perioperative period, tissue injury, inflammation, oxidative stress, and metabolic fluctuation increase membrane turnover and generate oxidized lipids and protein debris. Under these conditions, impaired extracellular chaperone support or weaker lipid transport can reduce the capacity of the brain to stabilize damaged membranes and clear stress-related material (Santos-García et al., 2025; Aikawa et al., 2021). Common-variant burden in *CLU* and *ABCA7* may therefore contribute to perioperative neurocognitive vulnerability by reducing lipid-linked repair capacity and by weakening debris handling during acute systemic stress (Duchateau et al., 2024; Santos-García et al., 2025; Aikawa et al., 2021).

### 5.2 Endocytosis, vesicle trafficking, and APP routing

A large part of non-*APOE* Alzheimer’s disease genetics converges on membrane trafficking, endocytosis, vesicle recycling, and intracellular cargo sorting (Kunkle et al., 2019; Jansen et al., 2019; Maninger et al., 2024; Szabo et al., 2022). Key genes in this network include *BIN1*, *PICALM*, *CD2AP*, and *SORL1*. These genes regulate how synaptic membranes are internalized and reshaped, how receptors are recycled, and how membrane-associated proteins move through endosomal and lysosomal pathways. Because synaptic function depends on constant membrane turnover and receptor repositioning, disruption in this network can impair neuronal signaling even before permanent structural injury appears.

*BIN1* encodes bridging integrator 1, a membrane-binding protein involved in membrane curvature and vesicle formation (Vandal et al., 2025a; Mishra et al., 2022). In neurons, *BIN1* is linked to endocytosis, cytoskeletal organization, calcium balance, and synaptic vesicle dynamics. It has also been connected to both amyloid-related and tau-related biology. Changes in *BIN1*-related function can alter membrane remodeling and intracellular trafficking, which may reduce the ability of neurons to maintain efficient synaptic communication during periods of physiologic stress.

*PICALM*, which encodes phosphatidylinositol-binding clathrin assembly protein, is a central component of clathrin-mediated endocytosis (Kunkle et al., 2019; Szabo et al., 2022; Mishra et al., 2022). It regulates receptor internalization, synaptic vesicle recycling, and movement of membrane proteins through trafficking pathways. It also influences autophagy-related transport and has been linked to amyloid precursor protein processing (Maninger et al., 2024). If *PICALM*-dependent trafficking is less efficient, neurons may clear damaged cargo more slowly and may recover less well after inflammatory activation or metabolic disruption.

*CD2AP* encodes an adaptor protein that links membrane trafficking to the actin cytoskeleton (Szabo et al., 2022; Dourlen et al., 2025; Zhang et al., 2024). It supports endocytosis, membrane stability, and vesicle movement. Because actin remodeling is essential for dendritic spine structure and receptor anchoring, *CD2AP*-related changes may alter the structural stability of synapses under acute stress. *SORL1*, which encodes sortilin-related receptor 1, regulates intracellular sorting of amyloid precursor protein and related cargo (Limone et al., 2022). Reduced SORL1 function shifts APP trafficking toward compartments that favor amyloidogenic processing, but its significance extends beyond amyloid generation. It also reflects the efficiency of intracellular cargo routing more broadly, including pathways needed for membrane protein turnover and receptor homeostasis (Kunkle et al., 2019; Limone et al., 2022).

Later perioperative neurocognitive phenotypes can emerge from reversible synaptic failure long before irreversible neurodegeneration is present (Jansen et al., 2019; Maninger et al., 2024; Mishra et al., 2022). Surgery, anesthesia, sleep disruption, inflammatory cytokines, and transient perfusion changes all place pressure on synaptic homeostasis. In that setting, efficient vesicle recycling and intracellular sorting become critical for preserving transmission. Common-variant burden across *BIN1*, *PICALM*, *CD2AP*, and *SORL1* may reduce this adaptive reserve and make the aging brain less able to maintain stable synaptic signaling after perioperative stress (Evered et al., 2018a).

### 5.3 Innate immunity and microglial signaling

A large group of Alzheimer’s disease risk loci acts through innate immune signaling and microglial state regulation (McFarland and Chakrabarty, 2022; Miao et al., 2023; Jorfi et al., 2023). The most relevant genes in this set include *TREM2*, *CD33*, *INPP5D*, *PLCG2*, and members of the *MS4A* family (McFarland and Chakrabarty, 2022; Miao et al., 2023; Jorfi et al., 2023; Hou et al., 2022). These loci are important because microglia control inflammatory sensing, phagocytosis, synaptic pruning, and the response to cellular injury (McFarland and Chakrabarty, 2022; Miao et al., 2023; Wu et al., 2025; Shi et al., 2025). In postoperative delirium and related perioperative neurocognitive states, neuroimmune activation is often a major short-term mechanism, so variation in this network has direct translational relevance (Yao et al., 2024; Paunikar and Chakole, 2024).

*TREM2* encodes triggering receptor expressed on myeloid cells 2, a receptor expressed mainly on microglia in the central nervous system (Hou et al., 2022; Zheng and Wang, 2025). It supports microglial survival, sensing of lipid-rich debris, phagocytic activity, and shifts into activated states (Shi et al., 2025; Yao et al., 2024; Zheng and Wang, 2025). Reduced *TREM2*-linked signaling can impair microglial clearance of damaged material and can alter how microglia respond to acute injury signals (Wu et al., 2025; Yao et al., 2024). This may change both the magnitude and the duration of neuroinflammatory activation after systemic stress (Wu et al., 2025; Yao et al., 2024).

*CD33* encodes a sialic acid-binding receptor expressed on myeloid cells. In the brain, *CD33* is associated with suppression of phagocytic activity and reduced uptake of amyloid-related material by microglia (McFarland and Chakrabarty, 2022; Miao et al., 2023; Jorfi et al., 2023; Akinluyi et al., 2026). Higher *CD33*-linked inhibitory tone may therefore limit debris clearance and prolong local inflammatory stress (Jorfi et al., 2023; Akinluyi et al., 2026). *INPP5D*, also known as *SHIP1*, is a phosphatase that modulates myeloid signaling pathways and can dampen downstream activation related to phagocytosis. Increased *INPP5D*-linked inhibitory signaling may further weaken clearance function (McFarland and Chakrabarty, 2022; Jorfi et al., 2023). *PLCG2* encodes phospholipase C gamma 2, which acts downstream of immune receptors in myeloid cells and influences calcium-linked signaling, activation-state transitions, and broader transcriptional responses (Bedford et al., 2025; Li et al., 2022). *MS4A*-family genes appear to affect receptor processing and immune membrane signaling, and part of their relevance may involve regulation of *TREM2*-related biology and microglial activation balance (Miao et al., 2023; Akinluyi et al., 2026).

Surgery triggers systemic cytokine release, endothelial activation, acute phase signaling, and interaction between circulating immune cells and the neurovascular interface. In patients with pre-existing genetic bias toward altered microglial activation or weaker phagocytic control, the same perioperative stress may produce a stronger or more prolonged inflammatory response inside the brain. This creates a plausible route from Alzheimer’s disease-related innate immune loci to postoperative delirium, especially in older adults who already have limited neuroimmune reserve (Yao et al., 2024; Paunikar and Chakole, 2024).

### 5.4 Complement signaling and opsonic clearance

*CR1* is one of the main complement-related loci linked to Alzheimer’s disease risk and remains highly relevant to perioperative neurocognitive injury (Daskoulidou et al., 2025; Daskoulidou et al., 2023; Veteleanu et al., 2023; Torvell et al., 2021). It encodes complement receptor 1, which binds complement-opsonized material and supports its capture and clearance (Daskoulidou et al., 2025; Daskoulidou et al., 2023). In the immune system, this receptor helps process tagged cellular debris and immune complexes. In the brain, complement signaling has a broader role because it also participates in synaptic tagging, microglial recognition of synaptic elements, and amplification of inflammatory signaling (Daskoulidou et al., 2023; Tenner and Petrisko, 2025; Dejanovic et al., 2022; Gomez-Arboledas et al., 2024; Ayyubova and Fazal, 2024; Batista et al., 2024).

Altered *CR1* function has been associated with reduced complement-mediated handling of amyloid-associated material (Daskoulidou et al., 2025; Veteleanu et al., 2023). This suggests that *CR1* variation may reduce extracellular immune clearance efficiency (Daskoulidou et al., 2025). In recent delirium-focused genetic and proteomic work, *CR1* was specifically highlighted as a candidate locus of interest, and plasma *CR1*-related signal showed nominal association (Leung et al., 2007; Raptis et al., 2026). That observation is biologically plausible because complement activation is increasingly recognized as a contributor to both acute and chronic synaptic dysfunction (Tenner and Petrisko, 2025; Dejanovic et al., 2022; Gomez-Arboledas et al., 2024; Ayyubova and Fazal, 2024; Batista et al., 2024).

During the perioperative period, tissue injury activates systemic inflammatory cascades and may increase complement activity (Paunikar and Chakole, 2024). If complement signaling becomes excessive, poorly regulated, or less effectively resolved, synaptic elements may be tagged inappropriately, and microglial pruning-like responses may increase (Dejanovic et al., 2022; Gomez-Arboledas et al., 2024; Ayyubova and Fazal, 2024; Batista et al., 2024). Even without permanent neurodegeneration, this can destabilize synaptic transmission and contribute to inattention, confusion, and delayed cognitive recovery (Gomez-Arboledas et al., 2024). In this way, *CR1*-related common-variant burden can reflect susceptibility to immune-mediated synaptic disturbance under acute surgical stress (Raptis et al., 2026; Paunikar and Chakole, 2024; Veteleanu et al., 2023).

### 5.5 Mitochondrial transport and oxidative stress

*TOMM40* is located adjacent to *APOE* on chromosome 19 and is often difficult to separate statistically because of linkage disequilibrium across the region (Kunkle et al., 2019; Jansen et al., 2019; Roses et al., 2010). For this reason, researchers should not place highly correlated *TOMM40*-and *APOE*-region variants into the same additive PRS without special handling, because this can count the same local signal more than once. A more robust strategy is to remove the extended *APOE*-region signal from the non-*APOE* PRS, model *APOE* genotype separately, and then test residual *TOMM40* effects with conditional regression, haplotype-based analysis, or fine-mapping after adjustment for *APOE* ε2/ε3/ε4 status (Ware et al., 2020; Jun et al., 2012). Even so, *TOMM40* has a biologically plausible role that is not limited to *APOE* tagging. It encodes a core component of the translocase of the outer mitochondrial membrane complex, which is required for import of nuclear-encoded proteins into mitochondria (Araiso et al., 2019; Pfanner et al., 2019; Chacinska et al., 2009). This process is essential for maintaining oxidative phosphorylation, calcium buffering, reactive oxygen species control, and mitochondrial stress responses (Araiso et al., 2019; Pfanner et al., 2019; Wang et al., 2020).

Neurons are highly dependent on mitochondrial stability because synaptic transmission, membrane repolarization, and intracellular ion homeostasis all require sustained ATP production (Devine and Kittler, 2018; Rangaraju et al., 2019). If mitochondrial protein import becomes less efficient, stress buffering capacity falls. This can make neurons more vulnerable to oxidative load, calcium dysregulation, and metabolic instability (Wang et al., 2020).

The perioperative setting places direct pressure on mitochondrial function. Anesthetic exposure, transient hypoxia, blood pressure fluctuation, inflammation, pain, and postoperative metabolic shifts all increase bioenergetic demand (Subramaniyan and Terrando, 2019; Vacas et al., 2014). In older brains, mitochondrial reserve is already reduced (Sun et al., 2016; Swerdlow, 2018). Genetic burden that affects *TOMM40*-linked pathways may therefore lower the threshold for cognitive decompensation under acute perioperative stress. In this context, *TOMM40* may index vulnerability to the energetic and oxidative dimension of perioperative neurocognitive injury, not only to classic amyloid-linked disease processes.

### 5.6 Blood–brain barrier and neurovascular vulnerability

The neurovascular system is another major point where Alzheimer’s disease genetics intersects with perioperative neurocognitive risk. Relevant loci include *EPHA1*, *CD2AP*, and the vascular effects associated with *APOE*, especially *APOE* ε4(111, 112). These genes influence the integrity of the neurovascular unit, which includes endothelial cells, pericytes, astrocytic end-feet, basement membrane components, and local immune signals (Kirchner et al., 2023; Vandal et al., 2025b; Cochran et al., 2015).

*EPHA1* encodes ephrin type-A receptor 1, a receptor tyrosine kinase involved in cell signaling, tissue organization, and membrane-related signaling. Functional studies have linked *EPHA1* to immune pathways, membrane trafficking, and blood–brain barrier stability (Owens et al., 2024). Changes in *EPHA1*-related signaling may alter endothelial responses during inflammation and may affect how well the barrier resists systemic stress. *CD2AP*, beyond its role in vesicle trafficking, also contributes to cytoskeletal support and membrane organization, which makes it relevant to barrier architecture as well as intracellular transport (Vandal et al., 2025b; Cochran et al., 2015). *APOE* ε4 adds a stronger and better-characterized vascular signal. Experimental work has linked *APOE4* to increased barrier permeability, altered astrocyte–vascular support, greater matrix-degrading activity, and reduced tight junction integrity (Kirchner et al., 2023; Reas et al., 2024).

Surgery stresses the blood–brain barrier through several linked mechanisms. Systemic inflammation activates endothelial cells. Hemodynamic instability changes perfusion pressure (Su et al., 2025). Hypoxia, infection, pain, and metabolic disturbance can further weaken barrier regulation. If the neurovascular unit is already less stable because of genetic burden, these perioperative stressors may push the barrier toward dysfunction more easily (Kirchner et al., 2023). Once permeability increases, peripheral cytokines, complement components, and immune cells can influence brain tissue more directly (Su et al., 2025). This can disturb neuronal signaling, amplify microglial activation, and increase the risk of postoperative delirium or delayed neurocognitive recovery (Kirchner et al., 2023; Owens et al., 2024; Reas et al., 2024).

## 6 Genotype-to-phenotype links and perioperative genomic model design

### 6.1 Biological pathways linking AD-related genetic burden to perioperative neurocognitive disorders

The connection between Alzheimer’s disease-related common-risk loci and perioperative neurocognitive disorders becomes clearer when the genetic signal is placed inside the biologic systems that are stressed during surgery (Kunkle et al., 2019; Bellenguez et al., 2022). A large part of this link runs through neuroimmune activation. *APOE* ε4, together with variation in *TREM2*, *CD33*, *INPP5D*, *PLCG2*, and *MS4A*-family genes, changes how microglia sense lipid debris, how strongly they respond to inflammatory input, and how efficiently they clear damaged synaptic material (Kunkle et al., 2019; Heneka et al., 2015). Surgical injury produces a strong systemic response that includes damage-associated molecular patterns, cytokines, complement activation, and endothelial signaling. These signals reach the neurovascular interface early after tissue trauma. In an older brain that already carries a higher inflammatory set point, microglia can enter a reactive state more quickly, release more inflammatory mediators, and remove synaptic debris less efficiently. This provides a biologically direct route to postoperative delirium, where inattention, fluctuating awareness, and acute network dysfunction develop over a short time window. Current reviews of PND increasingly place neuroinflammation at the center of this process, which is consistent with the way these loci are interpreted in Alzheimer’s disease genetics (Evered et al., 2018b; Hovens et al., 2014).

A large part of delayed neurocognitive recovery and postoperative neurocognitive disorder can also be understood through stress on synaptic membranes and intracellular trafficking. *BIN1*, *PICALM*, *CD2AP*, and *SORL1* regulate membrane curvature, receptor internalization, vesicle recycling, endosomal sorting, and cargo routing. These functions are essential for maintaining synaptic transmission when neurons are exposed to sleep disruption, pain, sedative drugs, fluctuating neurotransmitter tone, transient hypoperfusion, and inflammatory signaling (Hovens et al., 2014). During the perioperative period, synapses need rapid receptor turnover and stable membrane recycling to preserve network activity. If endocytosis, vesicle reuse, or endosomal sorting is already less efficient because of distributed common-variant burden, then a short-lived physiologic disturbance can more easily produce longer-lasting network instability (Harold et al., 2009; Small et al., 2005). That mechanism fits especially well with delayed neurocognitive recovery and later postoperative neurocognitive disorder, where the patient remains cognitively impaired after the acute delirium phase has passed (Hovens et al., 2014). The current PND framework recognizes these later phenotypes as distinct from delirium, which makes this trafficking-based interpretation more useful than treating all postoperative cognitive outcomes as one endpoint (Evered et al., 2018b; Hovens et al., 2014).

Neurovascular stress adds another direct path from genotype to perioperative phenotype. *APOE* ε4 is linked to increased blood–brain barrier fragility, and *EPHA1* and *CD2AP* are also connected to endothelial support and membrane architecture that can influence barrier stability. In this setting, clinically important dysfunction does not require a large infarct or visible structural lesion. Small increases in permeability can alter the ionic and inflammatory environment around neurons, expose brain tissue to circulating cytokines and complement proteins, and intensify local glial activation. Surgery places repeated pressure on the neurovascular unit through systemic inflammation, blood pressure fluctuation, hypoxia, infection, pain, and metabolic instability. In patients with weaker endothelial–pericyte–astrocyte coordination, these insults can produce subtle barrier leakage, impaired neuronal-glial coupling, and unstable network synchrony. This offers a strong biologic explanation for why some older adults show marked cognitive decompensation after operations that appear routine from a purely surgical point of view (Montagne et al., 2020).

**FIGURE 3:**
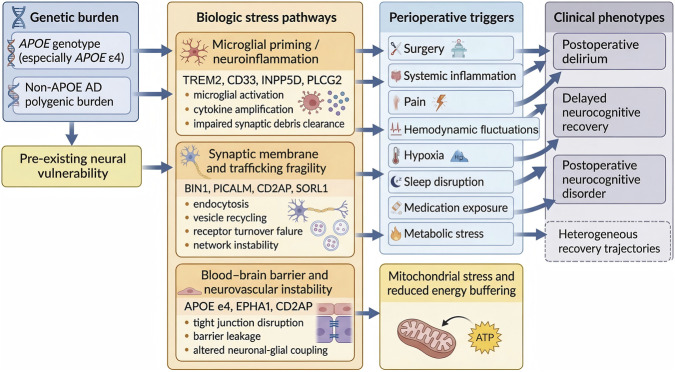
Mechanistic bridge from AD-related genetic burden to perioperative neurocognitive phenotypes. This schematic shows a layered framework linking Alzheimer’s disease-related genetic burden to perioperative neurocognitive phenotypes. In the left column, *APOE* genotype, especially *APOE* ε4, and non-*APOE* Alzheimer’s disease polygenic burden are shown as converging genetic inputs that contribute to pre-existing neural vulnerability before surgery. In the second column, this baseline vulnerability is translated into four major biologic stress pathways. The first pathway is microglial priming and neuroinflammation. It includes *TREM2*, *CD33*, *INPP5D*, *PLCG2*, and it is linked to altered microglial activation, cytokine amplification, and impaired clearance of synaptic debris. The second pathway is synaptic membrane and trafficking fragility. It includes *BIN1*, *PICALM*, *CD2AP*, and *SORL1*, and it reflects disrupted endocytosis, impaired vesicle recycling, failed receptor turnover, and broader network instability. The third pathway is blood–brain barrier and neurovascular instability. It includes *APOE* ε4, *EPHA1*, and *CD2AP*, and it highlights tight junction disruption, barrier leakage, and altered neuronal–glial coupling. The fourth pathway is mitochondrial stress and reduced energy buffering. It includes *TOMM40* and *APOE*-linked lipid imbalance, and it reflects ATP supply stress, oxidative load, and reduced metabolic resilience. In the third column, representative perioperative triggers are shown, including surgery, systemic inflammation, pain, hemodynamic fluctuations, hypoxia, sleep disruption, medication exposure, and metabolic stress. These insults interact with the vulnerable biologic pathways and worsen them. In the right column, the combined effects of these mechanisms are linked to postoperative delirium, delayed neurocognitive recovery, and postoperative neurocognitive disorder. These outcomes may occur in different patterns across patients, and recovery trajectories may vary after surgery. The figure shows how Alzheimer’s disease-related genetic architecture may be translated into clinically relevant perioperative cognitive phenotypes through biologic susceptibility pathways exposed by surgical stress.

Reduced mitochondrial buffering capacity is also a plausible part of the perioperative phenotype. *TOMM40*, *APOE*-linked lipid imbalance, and broader AD-related polygenic burden converge on weaker cellular resilience under metabolic stress. Neurons need sustained ATP production to preserve membrane gradients, calcium balance, synaptic transmission, and repair pathways. The perioperative period sharply increases energy demand through anesthetic exposure, inflammation, pain, transient hypoperfusion, and postoperative metabolic shifts. In that context, delirium can be viewed in part as a failure of network energy homeostasis under acute systemic stress. A higher AD-informed polygenic burden may therefore capture reduced resilience across lipid repair, oxidative control, and mitochondrial maintenance at the same time. That interpretation is more clinically useful than treating the PRS as a generic dementia score, because it places the signal inside the stress biology that is actually active during surgery and recovery (Area-Gomez et al., 2012) (Figure 3).

These pathway-level mechanisms are also likely to converge at the level of large-scale brain networks. Recent bidirectional Mendelian randomization work supports this systems-level view by linking resting-state network integrity and dementia risk in both directions (Zhu et al., 2025a). Although these data are not perioperative, they support the idea that surgical stress may unmask latent vulnerability by pushing already fragile networks beyond their adaptive range (Zhu et al., 2025a). Recent Mendelian randomization analyses also suggest that smoking-related traits are associated with higher Alzheimer’s disease risk (Chen et al., 2025). This further supports the view that modifiable exposures can interact with polygenic background and reduce perioperative brain resilience (Chen et al., 2025).

### 6.2 *APOE*-aware genomic modeling strategy

A clinically useful perioperative genomic model should be built around this biology, but it should not rely on genetics alone. The clearest entry point is explicit *APOE* genotyping. *APOE* should remain a separate variable because its coding structure, effect size, and biologic meaning are distinct from the rest of the common-variant background. The same rs429358 and rs7412 framework that defines ε2, ε3, and ε4 in dementia genetics also anchors the strongest common-variant delirium signal (Raptis et al., 2026). A model that keeps *APOE* separate allows direct estimation of whether risk is largely ε4-driven, which is easier to interpret than a single blended score. A broader non-*APOE* Alzheimer’s disease polygenic risk score can then be added to capture distributed burden across lipid transport, innate immunity, complement activity, endosomal trafficking, and mitochondrial stress. Keeping this component outside the *APOE* region makes calibration easier to assess and reduces the chance that the large *APOE* effect will mask smaller but still meaningful signals from other loci. Recent surgical biobank work supports the biologic and translational relevance of this *APOE*-aware structure by showing that *APOE*-ε4 carriage and Alzheimer’s disease polygenic burden are both associated with neurocognitive outcomes in surgical populations (Thedim et al., 2025; Raptis et al., 2026). However, stronger evidence is still needed from perioperative studies that compare *APOE*-aware genomic models directly against clinical-only models.

### 6.3 Integration with clinical variables, biomarkers, and perioperative workflow

The genomic layer becomes more useful when it is placed on top of a strong perioperative clinical model (Evered et al., 2018a; Marcantonio, 2017; Inouye et al., 2014). Age, baseline cognitive testing when available, frailty, prior stroke or transient ischemic attack, vascular disease burden, sleep disorder burden, education or other proxies of cognitive reserve, surgery complexity, ICU exposure risk, and medication profiles that increase delirium risk should remain in the base model. This point matters because PND is not caused by one risk source. It reflects the interaction between baseline brain vulnerability and perioperative stress (Evered et al., 2018a; Marcantonio, 2017; Inouye et al., 2014). Genomic data become clinically meaningful when they refine this baseline estimate, not when they are treated as an isolated signal (Evered et al., 2018a; Cerejeira et al., 2014). The same logic extends to biomarker integration. Stable genetic susceptibility and current biologic stress are different kinds of information. Plasma or serum markers of neuroaxonal injury and glial activation, such as neurofilament light chain, tau-related measures, and inflammatory readouts, can add a current-state layer that the genome alone cannot provide.

Current evidence for model improvement remains early and uneven. The clearest quantitative example comes from the delirium genetic and proteomic study by Raptis et al., in which adding 18 selected proteins to a basic model with age, sex, and BMI increased the AUC from 0.764 to 0.791, but this change was not significant. When *APOE*-ε4 status was added together with the proteins, the AUC increased from 0.764 to 0.794, and this improvement was significant (P = 0.049) (Raptis et al., 2026). By contrast, most perioperative cohort studies have focused on association rather than formal comparison against clinical-only prediction models (Leung et al., 2007; Vasunilashorn et al., 2015; Vasunilashorn et al., 2020; Thedim et al., 2025). For this reason, genomic information should presently be viewed as an adjunct layer within multimodal perioperative risk assessment, rather than as a replacement for standard clinical evaluation.

An important limitation of this framework is ancestry. Most large Alzheimer’s disease genome-wide association studies that underpin current Alzheimer’s disease polygenic risk scores were built mainly in European ancestry cohorts. For example, the large Bellenguez study was based on the European Alzheimer & Dementia Biobank and United Kingdom Biobank datasets, and recent multi-ancestry work has also noted that Alzheimer’s disease genome-wide association studies remain predominantly European ancestry in composition (Kunkle et al., 2019; Jansen et al., 2019). The current perioperative biobank evidence is also not ancestry-balanced. In the Mass General Brigham surgical biobank study, 86% of participants were of European ancestry (Thedim et al., 2025). This matters because polygenic risk score performance is not stable across ancestries. A recent transferability study showed that a European-derived Alzheimer’s disease polygenic score performed poorly in African ancestry populations, and the association weakened further as African ancestry increased (Sun et al., 2024). In a recent multi-ancestry Alzheimer’s disease polygenic risk score analysis, the score performed best in Europeans and more modestly in Caribbean Hispanic, African American, and Native American groups, while no significant association was observed in the South Asian and East Asian groups in that dataset (Schork and Elman, 2023). For this reason, any perioperative framework that uses Alzheimer’s disease-informed polygenic risk scores should be presented as ancestry-aware. It should require validation, calibration, and threshold assessment in the target population before clinical translation.

An additional barrier to implementation is ethical and practical governance. Preoperative *APOE* or Alzheimer’s disease polygenic risk testing should not be treated as routine surgical laboratory work. Current Alzheimer’s disease genetic counseling guidelines state that counseling is an integral part of the testing protocol, and current polygenic risk score guidance also emphasizes the need for appropriate counseling and informed consent before testing (Sun et al., 2024; Schork and Elman, 2023). For this reason, any future perioperative workflow would need a defined pathway for test ordering, interpretation, and result disclosure, with access to genetics expertise rather than *ad hoc* disclosure in the preoperative clinic. Consent should explain that *APOE* ε4 is a susceptibility marker with implications beyond perioperative risk, that polygenic risk scores are probabilistic rather than diagnostic, and that results may also have implications for family members (Sun et al., 2024; Schork and Elman, 2023). Risk communication in the preoperative setting should therefore use plain language, make uncertainty explicit, and allow patients to decide whether they wish to receive this information. Existing *APOE* disclosure frameworks support standardized education, assessment of psychological readiness, and structured post-test discussion, but these models were developed mainly in research settings and are not yet established as routine perioperative care (Romic et al., 2026). For this reason, the proposed perioperative genomic workflow should be viewed as a future implementation framework rather than a current standard of care.

For implementation, the output of the model should be linked to clinical action rather than presented as a raw genetic probability (Evered et al., 2018a; Fong et al., 2015; Robinson et al., 2009). If validated in future studies, a higher-risk result could be used to identify patients who may benefit from more structured delirium-prevention pathways, closer medication review, stronger sleep protection, family reorientation strategies, and earlier postoperative cognitive monitoring (Fong et al., 2015; Robinson et al., 2009). A lower-risk result may still support routine monitoring, even if it leads to less intensive intervention. In the future, this step may also support mechanism-based target prioritization rather than only general prevention bundles. Recent genetic causality work identified five potential delirium drug targets, including *C4BPA*, *A2M*, *GRIK4*, *C1R*, and *SUMF1*, and these targets were linked mainly to immune-related biology (Zhu et al., 2025b). In parallel, recent experimental work showed that pharmacologic activation of TERT attenuated hippocampal inflammation, improved neurogenesis-related signaling, and reduced cognitive deficits in a cigarette-smoke model (Zheng et al., 2026). Although these data are not yet perioperative treatment standards, they suggest that future precision perioperative medicine may move toward genetically informed therapeutic prioritization (Zhu et al., 2025b; Zheng et al., 2026).

Operational translation also depends on perioperative management itself, not only on the patient’s genomic profile. A recent trial protocol comparing etomidate-based and propofol-based total intravenous anaesthesia for postoperative quality of recovery highlights that anaesthetic choice may influence early recovery, although outcome data are still pending. This suggests that future genomic profiling may eventually be interpreted together with anaesthetic strategy, but current evidence does not support genotype-based selection of a specific agent (Zhu et al., 2025c). A brief systems-level view is also relevant, because occupational stress in anaesthesiologists has been associated with worse psychological health, which supports the broader idea that perioperative safety depends on the care environment as well as on patient-level biologic risk (Xu et al., 2025). At present, however, this step remains conceptual. Without a defined downstream care pathway and prospective validation, a perioperative polygenic risk score remains mainly an association tool rather than a fully translational tool (Evered et al., 2018a; Fong et al., 2015; Robinson et al., 2009).

### 6.4 Score architecture for perioperative risk stratification

The most straightforward score architecture is an *APOE*-aware dual-score design (Thedim et al., 2025; Raptis et al., 2026). In this structure, *APOE* is modeled categorically, with ε2 treated as relatively protective, ε3 as reference, and ε4 as higher risk (Thedim et al., 2025; Xu et al., 2024). Alongside that, a continuous non-*APOE* AD-PRS is calculated after removing the *APOE* region (Xu et al., 2024; Sofer et al., 2023). This separation is also statistically important, because it reduces the chance that correlated *APOE*- and *TOMM40*-region variants will be counted twice in a simple additive score and makes the non-*APOE* component easier to interpret (Ware et al., 2020). This creates four clinically interpretable combinations: low *APOE* burden with low non-*APOE* burden, high *APOE* burden with low non-*APOE* burden, low *APOE* burden with high non-*APOE* burden, and high *APOE* burden with high non-*APOE* burden (Sun et al., 2024). These groups are easy to understand in practice, and they allow direct testing of whether *APOE* and broader common-variant burden contribute partly distinct information (Raptis et al., 2026; Sofer et al., 2023). That pattern is already consistent with dementia modeling and is a logical fit for perioperative stratification (Thedim et al., 2025; Raptis et al., 2026).

**FIGURE 4:**
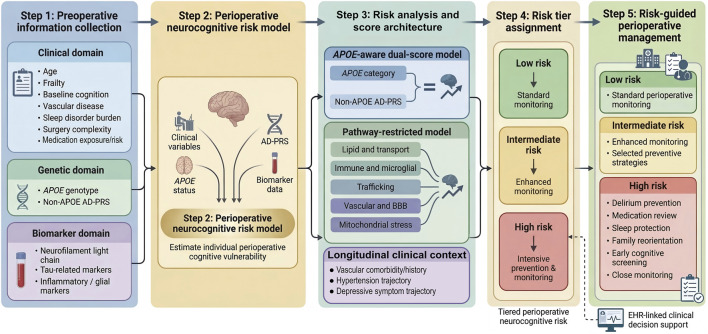
*APOE*-aware preoperative genomic risk stratification workflow for perioperative neurocognitive disorders. This schematic shows a conceptual five-step framework for *APOE*-aware preoperative genomic risk stratification in patients at risk for perioperative neurocognitive disorders (PND). Any future clinical use would require explicit consent, genetics-informed result interpretation, and structured communication of probabilistic risk. In Step 1, preoperative information is collected across three domains. The clinical domain includes age, frailty, baseline cognition, vascular disease, sleep disorder burden, surgery complexity, and medication exposure/risk. The genetic domain includes *APOE* genotype and non-*APOE* Alzheimer’s disease polygenic risk score (AD-PRS). The biomarker domain includes neurofilament light chain, tau-related markers, and inflammatory or glial markers. In Step 2, these data are combined in a central perioperative neurocognitive risk model. This model integrates clinical variables, *APOE* status, AD-PRS, and biomarker data to estimate individual perioperative cognitive vulnerability. In Step 3, the model output is organized using complementary score designs and interpreted in the context of longitudinal clinical history. These include an *APOE*-aware dual-score model, a pathway-restricted model, and clinical context from vascular comorbidity patterns and depressive-symptom trajectories. In Step 4, the model output is translated into low-, intermediate-, and high-risk tiers. In Step 5, the assigned risk tier could help guide perioperative management. In the future, this framework may also support mechanism-based therapeutic prioritization when targets supported by genetic or causal evidence become available. In addition, future implementation may need to consider modifiable perioperative factors such as anaesthetic strategy, rather than interpreting genomic risk in isolation. High-risk patients may receive a delirium prevention bundle, medication review, targeted preventive measures, sleep protection, family reorientation, early postoperative cognitive screening, and closer monitoring. Intermediate-risk patients may receive enhanced monitoring and selected preventive strategies. Low-risk patients may proceed with standard perioperative monitoring. The figure also highlights the possible use of electronic health record (EHR)-linked clinical decision support. This figure outlines a possible future route for combining genomics, biomarkers, and bedside clinical variables in perioperative cognitive risk assessment.

A more biologically explicit design uses pathway-restricted scores instead of one single global non-*APOE* score (Schork and Elman, 2023). In that approach, the common-variant burden is divided into function-based components (Schork and Elman, 2023; Romic et al., 2026). One score can represent lipid transport and extracellular handling through genes such as *CLU* and ABCA7(130). Another can represent innate immune and microglial signaling through *TREM2*, *CD33*, *INPP5D*, *PLCG2*, and *MS4A* loci (Romic et al., 2026). Another can represent endosomal and trafficking burden through *BIN1*, *PICALM*, *CD2AP*, and *SORL1* (Schork and Elman, 2023). A vascular and blood-brain barrier component can emphasize *EPHA1* and barrier-related loci, while a mitochondrial stress component can capture *TOMM40*-linked burden. This type of structure is attractive because it maps more directly onto perioperative subphenotypes. A stronger immune-pathway burden may align more closely with delirium driven by neuroinflammation (Romic et al., 2026). A stronger trafficking or lipid-related burden may fit patients with delayed neurocognitive recovery or later postoperative neurocognitive disorder after the acute postoperative phase (Schork and Elman, 2023). A stronger vascular burden may align with greater sensitivity to hemodynamic instability or barrier dysfunction. This kind of pathway-based design produces a PRS that is easier to interpret biologically and more consistent with mechanism-based perioperative translation (Andrews et al., 2023; Schork and Elman, 2023; Romic et al., 2026). This vascular domain should also be interpreted in light of longitudinal comorbidity patterns rather than as a static perioperative feature alone. Recent longitudinal evidence shows that new-onset hypertension is not linked to an immediate drop in cognition, but is linked to faster subsequent decline in global cognition, attention/calculation, and orientation in middle-aged and older adults (Zhu et al., 2025d). This pattern suggests that a single PRS value should be read together with the patient’s vascular trajectory and treatment history. In the same way, persistent depressive-symptom trajectories may also add clinical context to genomic risk. In a recent longitudinal ADNI study, persistently high depressive symptoms over 36 months after MCI diagnosis provided prognostic information for later conversion to Alzheimer’s disease (Ding et al., 2025). Although these data are not perioperative, they support the idea that longitudinal symptom trajectories may act as practical clinical proxies for underlying vulnerability during neurodegenerative progression. In this setting, pathway-based design is most useful when genetic burden is interpreted together with comorbidity history, symptom trajectory, and perioperative stress exposure (Figure 4).

## 7 Conclusion

*APOE* remains the most established genetic marker linked to perioperative neurocognitive vulnerability, but it does not capture the full range of baseline risk (Thedim et al., 2025; Raptis et al., 2026; Kirchner et al., 2023). Current delirium genetics and early perioperative cohort studies support a broader model in which *APOE* is combined with non-*APOE* Alzheimer’s disease polygenic burden (Thedim et al., 2025; Raptis et al., 2026). In this framework, genetic susceptibility is distributed across several biologic systems that are directly relevant to perioperative brain stress, including lipid transport, endosomal trafficking, microglial activation, complement-related immune signaling, mitochondrial stress handling, and blood–brain barrier stability (Miao et al., 2023; Zheng and Wang, 2025). These pathways shape how the aging brain responds to inflammation, vascular stress, metabolic fluctuation, oxidative load, sleep disruption, and other common perioperative insults (Paunikar and Chakole, 2024; Su et al., 2025). The clinical importance of this model is that it links genetic variation to specific forms of reduced brain resilience rather than treating perioperative neurocognitive disorders as a nonspecific extension of dementia risk (Andrews et al., 2023).

A practical translational approach is to use preoperative *APOE*-aware, AD-informed polygenic profiling as one layer within a larger perioperative risk model (Thedim et al., 2025; Raptis et al., 2026). In this setting, genomic information is most useful when it is integrated with baseline cognitive status, frailty, vascular comorbidity, surgery-related stress, circulating biomarkers, and structured perioperative workflows. This allows genetic susceptibility to be interpreted as part of a broader estimate of neurocognitive reserve and perioperative vulnerability (Paunikar and Chakole, 2024; Su et al., 2025). The current evidence does not support the use of polygenic profiling as a stand-alone diagnostic tool for perioperative neurocognitive disorders (Thedim et al., 2025). Instead, the available data support its use as a biologically informed risk-stratification layer, with early evidence of modest incremental value in selected models, while larger perioperative studies are still needed to define its added predictive value over clinical-only assessment and to determine how it should guide routine perioperative decision-making (Thedim et al., 2025; Paunikar and Chakole, 2024; Su et al., 2025).

## References

[B1] Aikawa T. Ren Y. Holm MREL Asmann Y. W. Alam A. Fitzgerald M. L. (2021). ABCA7 regulates brain fatty acid metabolism during LPS-induced acute inflammation. Front. Neurosci. 15, 647974. 10.3389/fnins.2021.647974 33897360 PMC8059705

[B2] AkinluyiE. T. Takahashi-YamashiroK. ConnollyM. G. PoonW. W. MacauleyM. S. (2026). Interplay between CD33 and TREM2 in Alzheimer'S disease: potential mechanistic insights into microglial function in amyloid pathology. ACS Chem. Neurosci. 17 (1), 62–76. 10.1021/acschemneuro.5c00805 41388352

[B3] Al-Ghraiybah N. F. Alkhalifa A. E. Itokazu Y. Farr T. O. Perez N. C. Ali H. (2026). Apolipoprotein e4 in Alzheimer'S disease: role in pathology, lipid metabolism, and drug treatment. Int. J. Mol. Sci. 27 (2), 1004. 10.3390/ijms27021004 41596649 PMC12842209

[B4] Andrews S. J. Renton A. E. Fulton-Howard B. Podlesny-Drabiniok A. Marcora E. Goate A. M. (2023). The complex genetic architecture of Alzheimer'S disease: novel insights and future directions. EBioMedicine 90, 104511. 10.1016/j.ebiom.2023.104511 36907103 PMC10024184

[B5] Araiso Y. Tsutsumi A. Qiu J. Imai K. Shiota T. Song J. (2019). Structure of the mitochondrial import gate reveals distinct preprotein paths. Nature 575 (7782), 395–401. 10.1038/s41586-019-1680-7 31600774

[B6] Area-Gomez E. Del Carmen Lara Castillo M. Tambini M. D. Guardia-Laguarta C. de Groof A. J. C. Madra M. (2012). Upregulated function of mitochondria-associated ER membranes in alzheimer disease. Embo J. 31 (21), 4106–4123. 10.1038/emboj.2012.202 22892566 PMC3492725

[B7] Ayyubova G. Fazal N. (2024). Beneficial versus detrimental effects of complement-microglial interactions in Alzheimer'S disease. Brain Sci. 14 (5), 434. 10.3390/brainsci14050434 38790413 PMC11119363

[B8] Batista A. F. Khan K. A. Papavergi MRAT Lemere C. A. (2024). The importance of complement-mediated immune signaling in Alzheimer'S disease pathogenesis. Int. J. Mol. Sci. 25 (2), 817. 10.3390/ijms25020817 38255891 PMC10815224

[B9] Bedford L. M. Tutrow K. D. Hooper K. Messenger E. J. Hernandez M. Lamb B. T. (2025). Alzheimer's disease-associated PLCG2 variants alter microglial state and function in human induced pluripotent stem cell-derived microglia-like cells. Alzheimers Dement. 21 (10), e70772. 10.1002/alz.70772 41066163 PMC12510136

[B10] Bellenguez C. L. E. Küçükali F. Jansen I. E. Kleineidam L. Moreno-Grau S. Amin N. (2022). New insights into the genetic etiology of Alzheimer'S disease and related dementias. Nat. Genet. 54 (4), 412–436. 10.1038/s41588-022-01024-z 35379992 PMC9005347

[B11] Belloy MCLE Napolioni V. Greicius M. D. (2019). A quarter century of APOE and Alzheimer'S disease: progress to date and the path forward. Neuron 101 (5), 820–838. 10.1016/j.neuron.2019.01.056 30844401 PMC6407643

[B12] Belloy M. E. Andrews S. J. Le Guen Y. Cuccaro M. Farrer L. A. Napolioni V. (2023). APOE genotype and alzheimer disease risk across age, sex, and population ancestry. JAMA Neurol. 80 (12), 1284–1294. 10.1001/jamaneurol.2023.3599 37930705 PMC10628838

[B13] Budny V. Ruminot I. N. Wybitul M. Treyer V. Barros L. F. Tackenberg C. (2025). Fueling the brain - the role of apolipoprotein e in brain energy metabolism and its implications for alzheimer's disease. Transl. Psychiatry 15 (1), 316. 10.1038/s41398-025-03550-w 40855008 PMC12379154

[B14] Cerejeira J. Lagarto L. S. Mukaetova-Ladinska E. B. (2014). The immunology of delirium. Neuroimmunomodulation 21 (2-3), 72–78. 10.1159/000356526 24557038

[B15] Chacinska A. Koehler C. M. Milenkovic D. Lithgow T. Pfanner N. (2009). Importing mitochondrial proteins: machineries and mechanisms. Cell 138 (4), 628–644. 10.1016/j.cell.2009.08.005 19703392 PMC4099469

[B16] Chandler H. L. Wheeler J. Escott-Price V. Murphy K. Lancaster T. M. (2025). Non-APOE variants predominately expressed in smooth muscle cells contribute to the influence of Alzheimer'S disease genetic risk on white matter hyperintensities. Alzheimers Dement. 21 (2), e14455. 10.1002/alz.14455 39737667 PMC11848156

[B17] Chen X. Wang L. Wang N. Li C. Hang H. Wu G. (2024). An apolipoprotein e receptor mimetic peptide decreases blood-brain barrier permeability following intracerebral hemorrhage by inhibiting the CypA/MMP-9 signaling pathway via LRP1 activation. Int. Immunopharmacol. 143 (Pt 3), 113007. 10.1016/j.intimp.2024.113007 39486173

[B18] Chen C. Zhu S. Zheng Z. Ding X. Shi W. Xia T. (2025). A genome-wide study on the genetic and causal effects of smoking in neurodegeneration. J. Transl. Med. 23 (1), 743. 10.1186/s12967-025-06688-9 40615926 PMC12228318

[B19] Clark K. Leung Y. Y. Lee W. P. Voight B. Wang L. S. (2022). Polygenic risk scores in Alzheimer'S disease genetics: methodology, applications, inclusion, and diversity. J. Alzheimers Dis. 89 (1), 1–12. 10.3233/JAD-220025 35848019 PMC9484091

[B20] Cochran J. N. Rush T. Buckingham S. C. Roberson E. D. (2015). The Alzheimer'S disease risk factor CD2AP maintains blood-brain barrier integrity. Hum. Mol. Genet. 24 (23), 6667–6674. 10.1093/hmg/ddv371 26358779 PMC4634373

[B21] Corder E. H. Saunders A. M. Strittmatter W. J. Schmechel D. E. Gaskell P. C. Small G. W. (1993). Gene dose of apolipoprotein e type 4 allele and the risk of alzheimer's disease in late onset families. Science 261 (5123), 921–923. 10.1126/science.8346443 8346443

[B22] Crehan H. Hardy J. Pocock J. (2012). Microglia, Alzheimer'S disease, and complement. Int. J. Alzheimers Dis. 2012, 983640. 10.1155/2012/983640 22957298 PMC3432348

[B23] Daskoulidou N. Shaw B. Torvell M. Watkins L. Cope E. L. Carpanini S. M. (2023). Complement receptor 1 is expressed on brain cells and in the human brain. Glia 71 (6), 1522–1535. 10.1002/glia.24355 36825534 PMC10953339

[B24] Daskoulidou N. Shaw B. Zelek W. M. Morgan B. P. (2025). The alzheimer's disease-associated complement receptor 1 variant confers risk by impacting glial phagocytosis. Alzheimers Dement. 21 (7), e70458. 10.1002/alz.70458 40631443 PMC12238831

[B25] Davis D. H. J. Muniz Terrera G. Keage H. Rahkonen T. Oinas M. Matthews F. E. (2012). Delirium is a strong risk factor for dementia in the oldest-old: a population-based cohort study. Brain 135 (Pt 9), 2809–2816. 10.1093/brain/aws190 22879644 PMC3437024

[B26] Deiner S. Silverstein J. H. (2009). Postoperative delirium and cognitive dysfunction. Br. J. Anaesth. 103 (Suppl. 1), i41–i46. 10.1093/bja/aep291 20007989 PMC2791855

[B27] Dejanovic B. Wu T. Tsai M. C. Graykowski D. Gandham V. D. Rose C. M. (2022). Complement c1q-dependent excitatory and inhibitory synapse elimination by astrocytes and microglia in Alzheimer'S disease mouse models. Nat. Aging 2 (9), 837–850. 10.1038/s43587-022-00281-1 37118504 PMC10154216

[B28] Devine M. J. Kittler J. T. (2018). Mitochondria at the neuronal presynapse in health and disease. Nat. Rev. Neurosci. 19 (2), 63–80. 10.1038/nrn.2017.170 29348666

[B29] Ding X. Zheng Z. Wang H. Shao Y. Zhu S. Ma Z. (2025). Persistent depressive-symptom trajectories predict conversion from mild cognitive impairment to Alzheimer'S disease: a longitudinal ADNI study. J. Affect Disord. 391, 120066. 10.1016/j.jad.2025.120066 40816362

[B30] Dourlen P. Kilinc D. Landrieu I. Chapuis J. Lambert J. C. (2025). BIN1 and Alzheimer'S disease: the tau connection. Trends Neurosci. 48 (5), 349–361. 10.1016/j.tins.2025.03.004 40268578

[B31] Duchateau L. Wawrzyniak N. Sleegers K. (2024). The ABC's of alzheimer risk gene ABCA7. Alzheimers Dement. 20 (5), 3629–3648. 10.1002/alz.13805 38556850 PMC11095487

[B32] Evered L. Silbert B. Knopman D. S. Scott D. A. DeKosky S. T. Rasmussen L. S. (2018a). Recommendations for the nomenclature of cognitive change associated with anaesthesia and surgery-2018. Br. J. Anaesth. 121 (5), 1005–1012. 10.1016/j.bja.2017.11.087 30336844 PMC7069032

[B33] Evered L. Silbert B. Knopman D. S. Scott D. A. DeKosky S. T. Rasmussen L. S. (2018b). Recommendations for the nomenclature of cognitive change associated with anaesthesia and surgery-20181. J. Alzheimers Dis. 66 (1), 1–10. 10.3233/JAD-189004 30347621

[B34] Fan K. H. Feingold E. Rosenthal S. L. Demirci F. Y. Ganguli M. Lopez O. L. (2020). Whole-exome sequencing analysis of Alzheimer'S disease in non-APOE*4 carriers. J. Alzheimers Dis. 76 (4), 1553–1565. 10.3233/JAD-200037 32651314 PMC7484092

[B35] Fong T. G. Davis D. Growdon M. E. Albuquerque A. Inouye S. K. (2015). The interface between delirium and dementia in elderly adults. Lancet Neurol. 14 (8), 823–832. 10.1016/S1474-4422(15)00101-5 26139023 PMC4535349

[B36] Fournier A. Krause R. Winterer G. Schneider R. (2015). Biomarkers of postoperative delirium and cognitive dysfunction. Front. Aging Neurosci. 7, 112. 10.3389/fnagi.2015.00112 26106326 PMC4460425

[B37] Ge T. Chen C. A. Y. Ni Y. Feng Y. C. A. Smoller J. W. (2019). Polygenic prediction via bayesian regression and continuous shrinkage priors. Nat. Commun. 10 (1), 1776. 10.1038/s41467-019-09718-5 30992449 PMC6467998

[B38] Gomez-Arboledas A. Fonseca M. I. Kramar E. Chu S. H. Schartz N. D. Selvan P. (2024). C5ar1 signaling promotes region- and age-dependent synaptic pruning in models of Alzheimer'S disease. Alzheimers Dement. 20 (3), 2173–2190. 10.1002/alz.13682 38278523 PMC10984438

[B39] Han X. Zhu S. Zhang H. Xia T. Gu X. (2026). Multiplex cerebrospinal fluid proteomics identifies biomarkers predicting neuropsychiatric symptom progression in mild cognitive impairment and Alzheimer'S disease. Faseb J. 40 (2), e71447. 10.1096/fj.202504014R 41553038

[B40] Harold D. Abraham R. Hollingworth P. Sims R. Gerrish A. Hamshere M. L. (2009). Genome-wide association study identifies variants at CLU and PICALM associated with Alzheimer'S disease. Nat. Genet. 41 (10), 1088–1093. 10.1038/ng.440 19734902 PMC2845877

[B41] He S. Xu Z. Han X. (2025). Lipidome disruption in Alzheimer'S disease brain: detection, pathological mechanisms, and therapeutic implications. Mol. Neurodegener. 20 (1), 11. 10.1186/s13024-025-00803-6 39871348 PMC11773937

[B42] Heneka M. T. Carson M. J. El Khoury J. Landreth G. E. Brosseron F. Feinstein D. L. (2015). Neuroinflammation in Alzheimer'S disease. Lancet Neurol. 14 (4), 388–405. 10.1016/S1474-4422(15)70016-5 25792098 PMC5909703

[B43] Hollingworth P. Harold D. Sims R. Gerrish A. Lambert J. C. Carrasquillo M. M. (2011). Common variants at ABCA7, MS4a6a/MS4a4e, EPHA1, CD33 and CD2AP are associated with Alzheimer'S disease. Nat. Genet. 43 (5), 429–435. 10.1038/ng.803 21460840 PMC3084173

[B44] Hou J. Chen Y. Grajales-Reyes G. Colonna M. (2022). TREM2 dependent and independent functions of microglia in Alzheimer'S disease. Mol. Neurodegener. 17 (1), 84. 10.1186/s13024-022-00588-y 36564824 PMC9783481

[B45] Hou T. Liu K. Fa W. Liu C. Zhu M. Liang X. (2024). Association of polygenic risk scores with Alzheimer'S disease and plasma biomarkers among chinese older adults: a community-based study. Alzheimers Dement. 20 (10), 6669–6681. 10.1002/alz.13924 39171679 PMC11485307

[B46] Hovens I. B. Schoemaker R. G. van der Zee E. A. Absalom A. R. Heineman E. van Leeuwen B. L. (2014). Postoperative cognitive dysfunction: involvement of neuroinflammation and neuronal functioning. Brain Behav. Immun. 38, 202–210. 10.1016/j.bbi.2014.02.002 24517920

[B47] Husain M. A. Laurent B. Plourde M. L. N. (2021). APOE and Alzheimer'S disease: from lipid transport to physiopathology and therapeutics. Front. Neurosci. 15, 630502. 10.3389/fnins.2021.630502 33679311 PMC7925634

[B48] Inouye S. K. Westendorp R. G. J. Saczynski J. S. (2014). Delirium in elderly people. Lancet 383 (9920), 911–922. 10.1016/S0140-6736(13)60688-1 23992774 PMC4120864

[B49] Islam S. Noorani A. Sun Y. Michikawa M. Zou K. (2025). Multi-functional role of apolipoprotein e in neurodegenerative diseases. Front. Aging Neurosci. 17, 1535280. 10.3389/fnagi.2025.1535280 39944166 PMC11813892

[B50] Jansen I. E. Savage J. E. Watanabe K. Bryois J. Williams D. M. Steinberg S. (2019). Genome-wide meta-analysis identifies new loci and functional pathways influencing Alzheimer'S disease risk. Nat. Genet. 51 (3), 404–413. 10.1038/s41588-018-0311-9 30617256 PMC6836675

[B51] Jiang W. Chen L. Girgenti M. J. Zhao H. (2024). Tuning parameters for polygenic risk score methods using GWAS summary statistics from training data. Nat. Commun. 15 (1), 24. 10.1038/s41467-023-44009-0 38169469 PMC10762162

[B52] Jorfi M. Maaser-Hecker A. Tanzi R. E. (2023). The neuroimmune axis of Alzheimer'S disease. Genome Med. 15 (1), 6. 10.1186/s13073-023-01155-w 36703235 PMC9878767

[B53] Jun G. Vardarajan B. N. Buros J. Yu C. E. Hawk M. V. Dombroski B. A. (2012). Comprehensive search for alzheimer disease susceptibility loci in the APOE region. Arch. Neurol. 69 (10), 1270–1279. 10.1001/archneurol.2012.2052 22869155 PMC3579659

[B54] Kauppi K. Rönnlund M. Nordin Adolfsson A. Pudas S. Adolfsson R. (2020). Effects of polygenic risk for Alzheimer'S disease on rate of cognitive decline in normal aging. Transl. Psychiatry 10 (1), 250. 10.1038/s41398-020-00934-y 32709845 PMC7381667

[B55] Kertai M. D. Mosley J. D. He J. Ramakrishnan A. Abdelmalak M. J. Hong Y. (2021). Predictive accuracy of a polygenic risk score for postoperative atrial fibrillation after cardiac surgery. Circ. Genom Precis. Med. 14 (2), e003269. 10.1161/CIRCGEN.120.003269 33647223 PMC8058298

[B56] Kirchner K. Garvert L. Kühn L. Bonk S. Grabe H. J. R. Van der Auwera S. (2023). Detrimental effects of ApoE ε4 on blood-brain barrier integrity and their potential implications on the pathogenesis of Alzheimer'S disease. Cells 12 (21), 2512. 10.3390/cells12212512 37947590 PMC10649078

[B57] Kunkle B. W. Grenier-Boley B. Sims R. Bis J. C. Damotte V. Naj A. C. (2019). Genetic meta-analysis of diagnosed Alzheimer'S disease identifies new risk loci and implicates aβ, tau, immunity and lipid processing. Nat. Genet. 51 (3), 414–430. 10.1038/s41588-019-0358-2 30820047 PMC6463297

[B58] Lake J. Warly Solsberg C. Kim J. J. Acosta-Uribe J. Makarious M. B. Li Z. (2023). Multi-ancestry meta-analysis and fine-mapping in Alzheimer'S disease. Mol. Psychiatry 28 (7), 3121–3132. 10.1038/s41380-023-02089-w 37198259 PMC10615750

[B59] Lambert J. C. Ibrahim-Verbaas C. A. Harold D. Naj A. C. Sims R. Bellenguez C. (2013). Meta-analysis of 74,046 individuals identifies 11 new susceptibility loci for Alzheimer'S disease. Nat. Genet. 45 (12), 1452–1458. 10.1038/ng.2802 24162737 PMC3896259

[B60] Laslo A. Laslo L. Elzmhel A. Ujlaki-Nagi A. A. Chinezu L. Ivănescu A. D. (2024). Pathways to alzheimer's disease: the intersecting roles of clusterin and apolipoprotein e in amyloid-β regulation and neuronal health. Pathophysiology 31 (4), 545–558. 10.3390/pathophysiology31040040 39449522 PMC11503414

[B61] Leung J. M. Sands L. P. Wang Y. Poon A. Kwok P. Y. Kane J. P. (2007). Apolipoprotein e e4 allele increases the risk of early postoperative delirium in older patients undergoing noncardiac surgery. Anesthesiology 107 (3), 406–411. 10.1097/01.anes.0000278905.07899.df 17721242

[B62] Li K. Ran B. Wang Y. Liu L. Li W. (2022). PLCγ2 impacts microglia-related effectors revealing variants and pathways important in Alzheimer'S disease. Front. Cell Dev. Biol. 10, 999061. 10.3389/fcell.2022.999061 36147734 PMC9485805

[B63] Li W. Shi Q. Bai R. Zeng J. Lin L. Dai X. (2025). Advances in research on the pathogenesis and signaling pathways associated with postoperative delirium (review). Mol. Med. Rep. 32 (2), 220. 10.3892/mmr.2025.13585 40476568 PMC12150672

[B64] Limone A. Veneruso I. D'Argenio V. Sarnataro D. (2022). Endosomal trafficking and related genetic underpinnings as a hub in Alzheimer'S disease. J. Cell Physiol. 237 (10), 3803–3815. 10.1002/jcp.30864 35994714 PMC9804649

[B65] Lin P. B. C. Holtzman D. M. (2024). Current insights into apolipoprotein e and the immune response in alzheimer's disease. Immunol. Rev. 327 (1), 43–52. 10.1111/imr.13414 39445515 PMC11578782

[B66] Liu A. Wang T. Yang L. Zhou Y. (2025). The APOE-microglia axis in Alzheimer'S disease: functional divergence and therapeutic perspectives-a narrative review. Brain Sci. 15 (7), 675. 10.3390/brainsci15070675 40722268 PMC12293602

[B67] Loomis S. J. Miller R. Castrillo-Viguera C. Umans K. Cheng W. O'Gorman J. (2024). Genome-wide association studies of ARIA from the aducanumab phase 3 ENGAGE and EMERGE studies. Neurology 102 (3), e207919. 10.1212/WNL.0000000000207919 38165296 PMC11097767

[B68] Maninger J. K. Nowak K. Goberdhan S. O'Donoghue R. Connor-Robson N. (2024). Cell type-specific functions of Alzheimer'S disease endocytic risk genes. Philos. Trans. R. Soc. Lond B Biol. Sci. 379 (1899), 20220378. 10.1098/rstb.2022.0378 38368934 PMC10874703

[B69] Marcantonio E. R. (2017). Delirium in hospitalized older adults. N. Engl. J. Med. 377 (15), 1456–1466. 10.1056/NEJMcp1605501 29020579 PMC5706782

[B70] McFarland K. N. Chakrabarty P. (2022). Microglia in Alzheimer'S disease: a key player in the transition between homeostasis and pathogenesis. Neurotherapeutics 19 (1), 186–208. 10.1007/s13311-021-01179-3 35286658 PMC9130399

[B71] Miao J. Ma H. Yang Y. Liao Y. Lin C. Zheng J. (2023). Microglia in Alzheimer'S disease: pathogenesis, mechanisms, and therapeutic potentials. Front. Aging Neurosci. 15, 1201982. 10.3389/fnagi.2023.1201982 37396657 PMC10309009

[B72] Milinkeviciute G. Green K. N. (2023). Clusterin/apolipoprotein j, its isoforms and Alzheimer'S disease. Front. Aging Neurosci. 15, 1167886. 10.3389/fnagi.2023.1167886 37122381 PMC10133478

[B73] Mishra S. Knupp A. Szabo M. P. Williams C. A. Kinoshita C. Hailey D. W. (2022). The alzheimer's gene SORL1 is a regulator of endosomal traffic and recycling in human neurons. Cell Mol. Life Sci. 79 (3), 162. 10.1007/s00018-022-04182-9 35226190 PMC8885486

[B74] Montagne A. Nation D. A. Sagare A. P. Barisano G. Sweeney M. D. Chakhoyan A. (2020). APOE4 leads to blood-brain barrier dysfunction predicting cognitive decline. Nature 581 (7806), 71–76. 10.1038/s41586-020-2247-3 32376954 PMC7250000

[B75] Najar J. Thorvaldsson V. Kern S. Skoog J. Waern M. Zetterberg H. (2023). Polygenic risk scores for Alzheimer'S disease in relation to cognitive change: a representative sample from the general population followed over 16 years. Neurobiol. Dis. 189, 106357. 10.1016/j.nbd.2023.106357 37977433

[B76] Owens H. A. Thorburn L. E. Walsby E. Moon O. R. Rizkallah P. Sherwani S. (2024). Alzheimer's disease-associated p460l variant of EphA1 dysregulates receptor activity and blood-brain barrier function. Alzheimers Dement. 20 (3), 2016–2033. 10.1002/alz.13603 38184788 PMC10984439

[B77] Paternò D. S. Via L. L. Putaggio A. Piccolo A. Scibilia G. Lentini M. (2026). Perioperative neurocognitive disorders: a narrative review of pathophysiology, prevention, and management strategies. J. Clin. Med. 15 (3), 1253. 10.3390/jcm15031253 41682934 PMC12897690

[B78] Paunikar S. Chakole V. (2024). Postoperative delirium and neurocognitive disorders: a comprehensive review of pathophysiology, risk factors, and management strategies. Cureus 16 (9), e68492. 10.7759/cureus.68492 39364454 PMC11447296

[B79] Pfanner N. Warscheid B. Wiedemann N. (2019). Mitochondrial proteins: from biogenesis to functional networks. Nat. Rev. Mol. Cell Biol. 20 (5), 267–284. 10.1038/s41580-018-0092-0 30626975 PMC6684368

[B80] Privé F. Arbel J. Vilhjálmsson B. J. (2021). LDpred2: better, faster, stronger. Bioinformatics 36 (22-23), 5424–5431. 10.1093/bioinformatics/btaa1029 33326037 PMC8016455

[B81] Rangaraju V. Lauterbach M. Schuman E. M. (2019). Spatially stable mitochondrial compartments fuel local translation during plasticity. Cell 176 (1-2), 73–84. 10.1016/j.cell.2018.12.013 30612742

[B82] Raptis V. Bhak Y. Cannings T. I. MacLullich A. M. J. Tenesa A. (2026). Dissecting the genetic and proteomic risk factors for delirium. Nat. Aging 6 (1), 235–251. 10.1038/s43587-025-01018-6 41286463 PMC12823428

[B83] Reas E. T. Solders S. K. Tsiknia A. Triebswetter C. Shen Q. Rivera C. S. (2024). APOE *ε*4-related blood-brain barrier breakdown is associated with microstructural abnormalities. Alzheimers Dement. 20 (12), 8615–8624. 10.1002/alz.14302 39411970 PMC11667544

[B84] Ren X. Huiqiao L. Wu Y. Zhang T. Chen P. Li L. (2025). Perioperative neurocognitive disorders: a comprehensive review of terminology, clinical implications, and future research directions. Front. Neurol. 16, 1526021. 10.3389/fneur.2025.1526021 40933055 PMC12418783

[B85] Riaz M. Huq A. Ryan J. Orchard S. G. Tiller J. Lockery J. (2021). Effect of APOE and a polygenic risk score on incident dementia and cognitive decline in a healthy older population. Aging Cell 20 (6), e13384. 10.1111/acel.13384 34041846 PMC8208779

[B86] Robinson T. N. Raeburn C. D. Tran Z. V. Angles E. M. Brenner L. A. Moss M. (2009). Postoperative delirium in the elderly: risk factors and outcomes. Ann. Surg. 249 (1), 173–178. 10.1097/SLA.0b013e31818e4776 19106695

[B87] Romic E. Karlsson I. Karalija N. Adolfsson A. N. Adolfsson R. Kauppi K. (2026). Pathway-based polygenic risk of Alzheimer'S disease highlights immune genes in cognitive decline. Alzheimers Dement. (N Y) 12 (1), e70209. 10.1002/trc2.70209 41669118 PMC12883303

[B88] Roses A. D. Lutz M. W. Amrine-Madsen H. Saunders A. M. Crenshaw D. G. Sundseth S. S. (2010). A TOMM40 variable-length polymorphism predicts the age of late-onset alzheimer'S disease. Pharmacogenomics J. 10 (5), 375–384. 10.1038/tpj.2009.69 20029386 PMC2946560

[B89] Sampatakakis S. N. Roma M. Scarmeas N. (2024). Subjective cognitive decline and genetic propensity for dementia beyond apolipoprotein ε(4): a systematic review. Curr. Issues Mol. Biol. 46 (3), 1975–1986. 10.3390/cimb46030129 38534745 PMC10969341

[B90] Santos-García I. Bascuñana P. Brackhan M. Villa M. A. Eiriz I. Brüning T. (2025). The ABC transporter a7 modulates neuroinflammation via NLRP3 inflammasome in Alzheimer'S disease mice. Alzheimers Res. Ther. 17 (1), 30. 10.1186/s13195-025-01673-2 39871385 PMC11773842

[B91] Schork N. J. Elman J. A. Alzheimer’s Disease Neuroimaging Initiative (2023). Pathway-specific polygenic risk scores correlate with clinical status and alzheimer’s disease-related biomarkers. J. Alzheimers Dis. 95 (3), 915–929. 10.3233/JAD-230548 37661888 PMC10697039

[B92] Sepulveda E. Adamis D. Franco J. G. Meagher D. Aranda S. Vilella E. (2021). The complex interaction of genetics and delirium: a systematic review and meta-analysis. Eur. Arch. Psychiatry Clin. Neurosci. 271 (5), 929–939. 10.1007/s00406-021-01255-x 33779822

[B93] Shi Q. Chowdhury S. Ma R. Le K. X. Hong S. Caldarone B. J. (2017). Complement C3 deficiency protects against neurodegeneration in aged plaque-rich APP/PS1 mice. Sci. Transl. Med. 9 (392). eaaf6295. 10.1126/scitranslmed.aaf6295 28566429 PMC6936623

[B94] Shi Q. Gutierrez R. A. Bhat M. A. (2025). Microglia, trem2, and neurodegeneration. Neuroscientist 31 (2), 159–176. 10.1177/10738584241254118 38769824 PMC11576490

[B95] Skoog I. Kern S. Najar J. Guerreiro R. Bras J. Waern M. (2021). A non-APOE polygenic risk score for Alzheimer'S disease is associated with cerebrospinal fluid neurofilament light in a representative sample of cognitively unimpaired 70-year olds. J. Gerontol. a Biol. Sci. Med. Sci. 76 (6), 983–990. 10.1093/gerona/glab030 33512503 PMC8140047

[B96] Small S. A. Kent K. Pierce A. Leung C. Kang M. S. Okada H. (2005). Model-guided microarray implicates the retromer complex in Alzheimer'S disease. Ann. Neurol. 58 (6), 909–919. 10.1002/ana.20667 16315276

[B97] Sofer T. Kurniansyah N. Granot-Hershkovitz E. Goodman M. O. Tarraf W. Broce I. (2023). A polygenic risk score for Alzheimer'S disease constructed using APOE-region variants has stronger association than APOE alleles with mild cognitive impairment in hispanic/latino adults in the u.s. Alzheimers Res. Ther. 15 (1), 146. 10.1186/s13195-023-01298-3 37649099 PMC10469805

[B98] Sprenger K. G. Lietzke E. E. Melchior J. T. Bruce K. D. (2025). Lipid and lipoprotein metabolism in microglia: alzheimer's disease mechanisms and interventions. J. Lipid Res. 66 (10), 100872. 10.1016/j.jlr.2025.100872 40769380 PMC12538436

[B99] Strickland M. R. Wang Z. Golden L. R. Wang H. Lu P. Ren Y. (2026). Lipidome and proteome of astrocyte and microglia ApoE lipoprotein reveal differences based on cell type and ApoE isoform. J. Lipid Res. 67, 101000. 10.1016/j.jlr.2026.101000 41692246 PMC12996666

[B100] Su Y. Chen Y. Zheng B. Huang Y. Liao Z. Zheng X. (2025). Structure and function of the blood-brain barrier in perioperative neurocognitive disorders. Front. Neurosci. 19, 1690354. 10.3389/fnins.2025.1690354 41585090 PMC12824458

[B101] Subramaniyan S. Terrando N. (2019). Neuroinflammation and perioperative neurocognitive disorders. Anesth. Analg. 128 (4), 781–788. 10.1213/ANE.0000000000004053 30883423 PMC6437083

[B102] Sun N. Youle R. J. Finkel T. (2016). The mitochondrial basis of aging. Mol. Cell 61 (5), 654–666. 10.1016/j.molcel.2016.01.028 26942670 PMC4779179

[B103] Sun M. He Q. Sun N. Han Q. Wang Y. Zhao H. (2024). Intrinsic capacity, polygenic risk score, APOE genotype, and risk of dementia: a prospective cohort study based on the UK biobank. Neurology 102 (12), e209452. 10.1212/WNL.0000000000209452 38843484

[B104] Supiyev A. Karlsson R. Wang Y. Koch E. Hägg S. Kauppi K. (2023). Independent role of Alzheimer'S disease genetics and c-reactive protein on cognitive ability in aging. Neurobiol. Aging 126, 103–112. 10.1016/j.neurobiolaging.2023.02.006 36965205

[B105] Swerdlow R. H. (2018). Mitochondria and mitochondrial cascades in Alzheimer'S disease. J. Alzheimers Dis. 62 (3), 1403–1416. 10.3233/JAD-170585 29036828 PMC5869994

[B106] Szabo M. P. Mishra S. Knupp A. Young J. E. (2022). The role of Alzheimer'S disease risk genes in endolysosomal pathways. Neurobiol. Dis. 162, 105576. 10.1016/j.nbd.2021.105576 34871734 PMC9071255

[B107] Tang A. S. Rankin K. P. Cerono G. Miramontes S. Mills H. Roger J. (2024). Leveraging electronic health records and knowledge networks for Alzheimer's disease prediction and sex-specific biological insights. Nat. Aging 4 (3), 379–395. 10.1038/s43587-024-00573-8 38383858 PMC10950787

[B108] Tenner A. J. Petrisko T. J. (2025). Knowing the enemy: strategic targeting of complement to treat alzheimer disease. Nat. Rev. Neurol. 21 (5), 250–264. 10.1038/s41582-025-01073-y 40128350 PMC12243624

[B109] Thedim M. Vacas S. (2024). Postoperative delirium and the older adult: untangling the confusion. J. Neurosurg. Anesthesiol. 36 (3), 184–189. 10.1097/ANA.0000000000000971 38683185 PMC11345733

[B110] Thedim M. Hu J. Maher M. Wiener-Kronish J. Saxena R. Vacas S. (2025). Perioperative polygenic and APOE-based genetic risk assessment for neurocognitive disorders: a biobank study. Br. J. Anaesth. 136, 1287–1293. 10.1016/j.bja.2025.05.014 40562635 PMC13168963

[B111] Thedim M. Li H. Formanek A. Wiener-Kronish J. Saxena R. Vacas S. (2026). Genetic and epigenetic insights into perioperative neurocognitive disorders: a narrative review. Br. J. Anaesth. 136, 1226–1234. 10.1016/j.bja.2025.12.046 41620312 PMC12997105

[B112] Tomassen J. den Braber A. van der Lee S. J. Reus L. M. Konijnenberg E. Carter S. F. (2022). Amyloid-β and APOE genotype predict memory decline in cognitively unimpaired older individuals independently of Alzheimer'S disease polygenic risk score. BMC Neurol. 22 (1), 484. 10.1186/s12883-022-02925-6 36522743 PMC9753236

[B113] Torvell M. Carpanini S. M. Daskoulidou N. Byrne R. A. J. Sims R. Morgan B. P. (2021). Genetic insights into the impact of complement in Alzheimer'S disease. Genes (Basel) 12 (12), 1990. 10.3390/genes12121990 34946939 PMC8702080

[B114] Troutwine B. R. Hamid L. Lysaker C. R. Strope T. A. Wilkins H. M. (2022). Apolipoprotein e and alzheimer's disease. Acta Pharm. Sin. B 12 (2), 496–510. 10.1016/j.apsb.2021.10.002 35256931 PMC8897057

[B115] Vacas S. Degos V. Tracey K. J. Maze M. (2014). High-mobility group box 1 protein initiates postoperative cognitive decline by engaging bone marrow-derived macrophages. Anesthesiology 120 (5), 1160–1167. 10.1097/ALN.0000000000000045 24162463 PMC3999217

[B116] Vandal M. Janmaleki M. Rea I. Gunn C. Hirai S. Biernaskie J. (2025a). CD2AP at the junction of nephropathy and Alzheimer'S disease. Mol. Neurodegener. 20 (1), 63. 10.1186/s13024-025-00852-x 40462155 PMC12135550

[B117] Vandal M. N. Institoris A. Reveret L. Korin B. Gunn C. Hirai S. (2025b). Loss of endothelial CD2AP causes sex-dependent cerebrovascular dysfunction. Neuron 113 (6), 876–895. 10.1016/j.neuron.2025.01.006 39892386

[B118] Vasiljevic E. Koscik R. L. Jonaitis E. Betthauser T. Johnson S. C. Engelman C. D. (2023). Cognitive trajectories diverge by genetic risk in a preclinical longitudinal cohort. Alzheimers Dement. 19 (7), 3108–3118. 10.1002/alz.12920 36723444 PMC10390653

[B119] Vasunilashorn S. Ngo L. Kosar C. M. Fong T. G. Jones R. N. Inouye S. K. (2015). Does apolipoprotein e genotype increase risk of postoperative delirium? Am. J. Geriatr. Psychiatry 23 (10), 1029–1037. 10.1016/j.jagp.2014.12.192 26238230 PMC4591079

[B120] Vasunilashorn S. M. Ngo L. H. Inouye S. K. Fong T. G. Jones R. N. Dillon S. T. (2020). Apolipoprotein e genotype and the association between c-reactive protein and postoperative delirium: importance of gene-protein interactions. Alzheimers Dement. 16 (3), 572–580. 10.1016/j.jalz.2019.09.080 31761478 PMC7086383

[B121] Veteleanu A. Stevenson-Hoare J. Keat S. Daskoulidou N. Zetterberg H. Heslegrave A. (2023). Alzheimer's disease-associated complement gene variants influence plasma complement protein levels. J. Neuroinflammation 20 (1), 169. 10.1186/s12974-023-02850-6 37480051 PMC10362776

[B122] Vromen E. M. Del Campo Milán M. Scheltens P. Teunissen C. E. Visser P. J. Tijms B. M. (2022). CSF proteomic signature predicts progression to Alzheimer'S disease dementia. Alzheimers Dement. (N Y) 8 (1), e12240. 10.1002/trc2.12240 35229020 PMC8864445

[B123] Wang W. Zhao F. Ma X. Perry G. Zhu X. (2020). Mitochondria dysfunction in the pathogenesis of Alzheimer'S disease: recent advances. Mol. Neurodegener. 15 (1), 30. 10.1186/s13024-020-00376-6 32471464 PMC7257174

[B124] Ware E. B. Faul J. D. Mitchell C. M. Bakulski K. M. (2020). Considering the APOE locus in Alzheimer'S disease polygenic scores in the health and retirement study: a longitudinal panel study. BMC Med. Genomics 13 (1), 164. 10.1186/s12920-020-00815-9 33143703 PMC7607711

[B125] Wu Z. Yu S. Tian D. Cheng L. Jing J. (2025). Microglial TREM2 and cognitive impairment: insights from Alzheimer'S disease with implications for spinal cord injury and AI-assisted therapeutics. Front. Cell Neurosci. 19, 1705069. 10.3389/fncel.2025.1705069 41280332 PMC12634590

[B126] Xia Z. Prescott E. E. Urbanek A. Wareing H. E. King M. C. Olerinyova A. (2024). Co-aggregation with apolipoprotein e modulates the function of amyloid-β in alzheimer's disease. Nat. Commun. 15 (1), 4695. 10.1038/s41467-024-49028-z 38824138 PMC11144216

[B127] Xu Y. Sun Z. Jonaitis E. Deming Y. Lu Q. Johnson S. C. (2024). Apolipoprotein E moderates the association between non-APOE polygenic risk score for Alzheimer's disease and aging on preclinical cognitive function. Alzheimers Dement. 20 (2), 1063–1075. 10.1101/2023.06.09.23291215 37858606 PMC10916952

[B128] Xu R. Chen W. Wang J. Ding X. Zhu S. Han X. (2025). Effects of occupational stress on the distribution of circulating t lymphocyte subsets and psychological health of young anaesthesiologists. BMC Psychol. 13 (1), 1358. 10.1186/s40359-025-03696-8 41388323 PMC12699846

[B129] Yang Y. Jung K. J. Kwak Y. T. (2025). The relationship between postoperative delirium and plasma amyloid beta oligomer. Sci. Rep. 15 (1), 13147. 10.1038/s41598-025-97577-0 40240804 PMC12003799

[B130] Yao Y. Hu L. Li D. Wang Y. Pan J. Fan D. (2024). Perioperative enriched environment attenuates postoperative cognitive dysfunction by upregulating microglia TREM2 *via* PI3k/akt pathway in mouse model of ischemic stroke. Front. Neurosci. 18, 1520710. 10.3389/fnins.2024.1520710 39758888 PMC11695310

[B131] Yuste-Checa P. Carvajal A. I. Mi C. Paatz S. Hartl F. U. Bracher A. (2025). Structural analyses define the molecular basis of clusterin chaperone function. Nat. Struct. Mol. Biol. 32 (10), 2035–2045. 10.1038/s41594-025-01631-4 40781479 PMC12527946

[B132] Zhang L. Huang L. Zhou Y. Meng J. Zhang L. Zhou Y. (2024). Microglial CD2AP deficiency exerts protection in an Alzheimer'S disease model of amyloidosis. Mol. Neurodegener. 19 (1), 95. 10.1186/s13024-024-00789-7 39695808 PMC11658232

[B133] Zhao X. Li Y. Zhang S. Sudwarts A. Zhang H. Kozlova A. (2025). Alzheimer's disease protective allele of clusterin modulates neuronal excitability through lipid-droplet-mediated neuron-glia communication. Mol. Neurodegener. 20 (1), 51. 10.1186/s13024-025-00840-1 40319306 PMC12049787

[B134] Zheng Q. Wang X. (2025). Alzheimer's disease: insights into pathology, molecular mechanisms, and therapy. Protein Cell 16 (2), 83–120. 10.1093/procel/pwae026 38733347 PMC11786724

[B135] Zheng Z. Chen C. Zhu S. Zhu X. Tu H. Ding X. (2026). TERT activator compound alleviates cigarette smoke-induced cognitive deficits by modulating hippocampal inflammation and neurogenesis: a comprehensive study integrating mendelian randomization. Exp. Neurol. 396, 115543. 10.1016/j.expneurol.2025.115543 41197759

[B136] Zhu S. Ding X. Bo J. Shi W. Xia T. Gu X. (2025a). Brain network connectivity and dementia risk: a bidirectional mendelian randomisation perspective. Neuroimage Clin. 48, 103913. 10.1016/j.nicl.2025.103913 41297292 PMC12685556

[B137] Zhu S. Ding X. Bo J. Xia T. Gu X. (2025b). Novel drug targets for delirium based on genetic causality. J. Affect Disord. 378, 128–137. 10.1016/j.jad.2025.02.095 40023257

[B138] Zhu S. Wu M. Ding X. Zhang W. Xia T. Bo J. (2025c). Effects of etomidate versus propofol for total intravenous anaesthesia on postoperative quality of recovery in patients undergoing day-case laparoscopic cholecystectomy: protocol for a multicentre, randomised controlled non-inferiority trial. BMJ Open 15 (9), e098584. 10.1136/bmjopen-2024-098584 40912714 PMC12414158

[B139] Zhu S. Bo J. Xia T. Gu X. (2025d). Temporal patterns of cognitive decline after hypertension onset among middle-aged and older adults in China. Sci. Rep. 15 (1), 16300. 10.1038/s41598-025-98267-7 40348933 PMC12065796

